# Supplementary material for: Molecular Signatures Related to Inflammation and Angiogenesis in Patients with Lower Extremity Artery Disease, Abdominal Aortic Aneurysm, and Varicose Veins: Shared and Distinct Pathways
Source: Int J Mol Sci. 2025 Sep 9;26(18):8786. doi: 10.3390/ijms26188786 (PMC12469879; doi:10.3390/ijms26188786)
Supplement: Supplementary file 1 [file ijms-26-08786-s001.zip › Supplementary Materials - Expanded Materials and Methods.pdf]

EXPANDED MATERIALS AND METHODS

Molecular Signatures related to Inflammation and Angiogenesis in Patients with Lower Extremity Artery Disease, Abdominal Aortic Aneurysm, and Varicose Veins: Shared and Distinct Pathways.

Daniel Zalewski [1,*], Paulina Chmiel [2], Przemysław Kołodziej [1], Marcin Feldo [3], Andrzej Stępniewski [4], Marta Ziaja-Sołtys [1], Joanna Łuszczak [1], Agata Stanek [5], Janusz Kocki [6] and Anna Bogucka-Kocka [1]

[1] Chair and Department of Biology and Genetics, Medical University of Lublin, 4a Chodźki St., 20-093 Lublin, Poland; daniel.piotr.zalewski@gmail.com (D.Z.); przemyslaw.kolodziej@umlub.edu.pl (P.K.); marta.ziaja-soltys@umlub.edu.pl (M.Z.-S.); joanna.luszczak@umlub.edu.pl (J.Ł.); anna.kocka@umlub.edu.pl (A.B-K.)

[2] Randox Laboratories Ltd., Poznańska St., 00-680 Warszawa, Poland; pachmiel13@gmail.com (P.Ch.)

[3] Chair and Department of Vascular Surgery and Angiology, Medical University of Lublin, 11 Staszica St., 20-081 Lublin, Poland; martinf@interia.pl (M.F.)

[4] ECOTECH-COMPLEX Analytical and Programme Centre for Advanced Environmentally Friendly Technologies, University of Marie Curie-Skłodowska, 39 Głęboka St., 20-612 Lublin, Poland; astep@ipan.lublin.pl (An.S.)

[5] Department of Internal Medicine, Metabolic Diseases and Angiology, Faculty of Health Sciences in Katowice, Medical University of Silesia, Ziołowa 45/47 St., 40-635 Katowice, Poland; astanek@sum.edu.pl (Ag.S.)

[6] Department of Clinical Genetics, Chair of Medical Genetics, Medical University of Lublin, 11 Radziwiłłowska St., 20-080 Lublin, Poland; janusz.kocki@umlub.edu.pl (J.K.)

* Correspondence: daniel.piotr.zalewski@gmail.com; daniel.zalewski@umlub.edu.pl; Tel.: +48-81-448-7236

Qualification of the studies participants

The study was performed using four previously obtained and publicly available datasets:

- Gene expression and protein plasma levels dataset for AAA group (https://doi.org/10.6084/m9.figshare.23791485.v1),
- Gene expression and protein plasma levels dataset for VV group (https://doi.org/10.6084/m9.figshare.26065462.v1),
- Gene expression and protein plasma levels dataset for LEAD group (access link will be provided upon acceptance),
- RNA-seq dataset (https://doi.org/10.6084/m9.figshare.14252897.v1),
- miRNA-seq dataset (https://doi.org/10.6084/m9.figshare.19446851.v2).

These datasets contains information about the expression of miRNAs and genes, as well as plasma protein levels in groups of patients with lower extremity artery disease (LEAD), abdominal aortic aneurysm (AAA), and varicose veins (VV). The studies, from which the datasets were derived, were conducted in compliance with the ethical principles outlined in the Declaration of Helsinki, and was approved by the Bioethics Committee of the Medical University of Lublin (approvals No. KE-0254/341/2015, KE-0254/148/2021, and KE-0254/227/2021). Informed and signed consent was obtained from all study subjects.

The studies participants were qualified in the Chair and Department of Vascular Surgery and Angiology of the Medical University of Lublin, based on the established inclusion criteria. The subjects enrolled in this study underwent comprehensive diagnostic procedures including medical interviews, physical examinations, and vascular imaging. The LEAD group comprised individuals with atherosclerotic plaques localized in the femoral, iliac, or popliteal arteries, classified with a Trans-Atlantic Inter-Society Consensus score of B or C, and Rutherford category 2 or 3 (claudication without critical ischemia or tissue loss). The AAA group consisted of patients who underwent preoperative aneurysm surveillance and were diagnosed with abdominal aortic aneurysm measuring 5.5 to 7.8 cm in diameter. The VV group included patients diagnosed with VV according to the CEAP (Clinical, Etiology, Anatomic, Pathophysiology) classification as follows: C2 (clinical presentation of varicose veins), As (presence of symptoms in superficial veins), Ep (primary etiology), and Pr (reflux as a pathophysiological mechanism).

The exclusion criteria common to all groups were myocardial infarction, stroke, type 2 diabetes mellitus, and pregnancy.

Venous blood samples were collected from the enrolled individuals and processed to separate plasma and peripheral blood mononuclear cells (PBMC) using standard and gradient centrifugation techniques, respectively.

Gene expression dataset

Gene expression datasets contain information about the expression of 18 genes (*ANGPT1*, *ANGPT2*, *CCL2*, *CCL5*, *CSF2*, *CXCL8*, *FGF2*, *IL1A*, *IL1B*, *IL6*, *PDGFA*, *PDGFB*, *TGFA*,

TGFB1, *TNF*, *VEGFA*, *VEGFB*, and *VEGFC*) in PBMC samples from 40 patients with LEAD (LEAD group), 40 patients with AAA (AAA group), and 40 patients with VV (VV group).

PBMC samples isolated from blood were subjected to total RNA isolation using TRI Reagent Solution (Ambion, Austin, TX, USA). Total RNA was reverse transcribed into complementary DNA (cDNA) using the High Capacity cDNA Reverse Transcription Kit (Applied Biosystems, Foster City, CA, USA). Real-time PCR reactions were subsequently prepared in 96-well plates using TaqMan Gene Expression Master Mix (Applied Biosystems, Foster City, CA, USA) and TaqMan Gene Expression Assays specific to target genes (Applied Biosystems, Foster City, CA, USA). Information regarding the assays used is provided in Table S15. The amplification reaction was carried out using the 7900HT Real-Time Fast System according to the manufacturer's instructions, and Ct values were determined using ExpressionSuite v1.3 software (Life Technologies Corporation, Carlsbad, CA, USA). The gene expression levels were quantified as cycle threshold (Ct) values, which represent the number of amplification cycle required for the fluorescence signal to intersect a threshold line defining the linear phase of signal amplification.

Protein plasma levels datasets

Protein plasma levels datasets are a part of gene expression datasets and contain information about the plasma concentrations of six proteins (ANGPT-1, ANGPT-2, TGF-alpha, TGF-beta 1, VEGF-A, and VEGF-C) in 40 patients with LEAD, 40 patients with AAA, and 40 patients with VV, the same as for gene expression dataset.

ELISA experiments were performed using commercially available ELISA kits purchased from Biorbyt (Cambridge, United Kingdom), according to the manufacturer's instructions (Table S16). The prepared ELISA plates were read at appropriate wavelengths using a Synergy H1 microplate reader (BioTek, Winooski, VT, USA). The absorbance values were determined using Gen5 version 3.10 software (BioTek, Winooski, VT, USA). Protein concentrations in the samples were calculated by interpolation from the standard curve and adjusted for the dilution factor when necessary.

RNA-seq dataset

This dataset contains the expression profiles of 55,765 genes in PBMC samples from eight patients with LEAD, seven patients with AAA, and seven patients with VV.

Total RNA was isolated from PBMC samples using TRI Reagent Solution (Applied Biosystems, Foster City, CA, USA), according to the manufacturer's instructions. Transcriptome libraries were prepared using the Ion Total RNA-Seq Kit v2, Magnetic Bead Cleanup Module kit, and Ion Xpress RNA-Seq Barcode 01-16 Kit (all Life Technologies, Carlsbad, CA, USA). The prepared libraries were sequenced on Ion 540 chips using Ion S5 XL System (Thermo Fisher Scientific, Waltham, MA, USA). Raw sequences were aligned to 55,765 genes of hg19 human genome and a matrix with raw reads counts calculated for each gene in each studied sample was generated.

miRNA-seq dataset

This dataset contains the expression profiles of 2792 miRNA transcripts in PBMC samples from 40 patients with LEAD, 28 patients with AAA, and 34 patients with VV.

For miRNA experiments, small RNA fractions were isolated from PBMC samples using the MirVana microRNA Isolation Kit (Ambion, Austin, TX, USA). Subsequently, small RNA samples were subjected to miRNA libraries preparation using the Ion Total RNA-Seq Kit v2, Magnetic Bead Cleanup Module kit, and Ion Xpress RNA-Seq Barcode 01-16 Kit (Life Technologies, Carlsbad, CA, USA). Libraries were sequenced on the Ion 540 chips (Life Technologies) using the Ion S5 XL System (Thermo Fisher Scientific, Waltham, MA, USA). Raw sequences were aligned to 2792 human miRNAs from miRBase v21 (http://www.mirbase.org) using the Torrent Suite Software v5.0.4. and the Ion Torrent Small RNA Plugin v5.0.5r3 plugin (Thermo Fisher Scientific, Waltham, MA, USA).
