# Supplementary material for: Molecular Signatures Related to Inflammation and Angiogenesis in Patients with Lower Extremity Artery Disease, Abdominal Aortic Aneurysm, and Varicose Veins: Shared and Distinct Pathways
Source: Int J Mol Sci. 2025 Sep 9;26(18):8786. doi: 10.3390/ijms26188786 (PMC12469879; doi:10.3390/ijms26188786)
Supplement: Supplementary file 1 [file ijms-26-08786-s001.zip › Supplementary Materials - Supplementary Figures.pdf]

SUPPLEMENTARY FIGURES (Figures S1-S34)

Molecular Signatures related to Inflammation and Angiogenesis in Patients with Lower Extremity Artery Disease, Abdominal Aortic Aneurysm, and Varicose Veins: Shared and Distinct Pathways.

Daniel Zalewski [1,*], Paulina Chmiel [2], Przemysław Kołodziej [1], Marcin Feldo [3], Andrzej Stępniewski [4], Marta Ziaja-Sołtys [1], Joanna Łuszczak [1], Agata Stanek [5], Janusz Kocki [6] and Anna Bogucka-Kocka [1]

[1] Chair and Department of Biology and Genetics, Medical University of Lublin, 4a Chodźki St., 20-093 Lublin, Poland; daniel.piotr.zalewski@gmail.com (D.Z.); przemyslaw.kolodziej@umlub.edu.pl (P.K.); marta.ziaja-soltys@umlub.edu.pl (M.Z.-S.); joanna.luszczak@umlub.edu.pl (J.Ł.); anna.kocka@umlub.edu.pl (A.B-K.)

[2] Randox Laboratories Ltd., Poznańska St., 00-680 Warszawa, Poland; pachmiel13@gmail.com (P.Ch.)

[3] Chair and Department of Vascular Surgery and Angiology, Medical University of Lublin, 11 Staszica St., 20-081 Lublin, Poland; martinf@interia.pl (M.F.)

[4] ECOTECH-COMPLEX Analytical and Programme Centre for Advanced Environmentally Friendly Technologies, University of Marie Curie-Skłodowska, 39 Głęboka St., 20-612 Lublin, Poland; astep@ipan.lublin.pl (An.S.)

[5] Department of Internal Medicine, Metabolic Diseases and Angiology, Faculty of Health Sciences in Katowice, Medical University of Silesia, Ziołowa 45/47 St., 40-635 Katowice, Poland; astanek@sum.edu.pl (Ag.S.)

[6] Department of Clinical Genetics, Chair of Medical Genetics, Medical University of Lublin, 11 Radziwiłłowska St., 20-080 Lublin, Poland; janusz.kocki@umlub.edu.pl (J.K.)

* Correspondence: daniel.piotr.zalewski@gmail.com; daniel.zalewski@umlub.edu.pl; Tel.: +48-81-448-7236

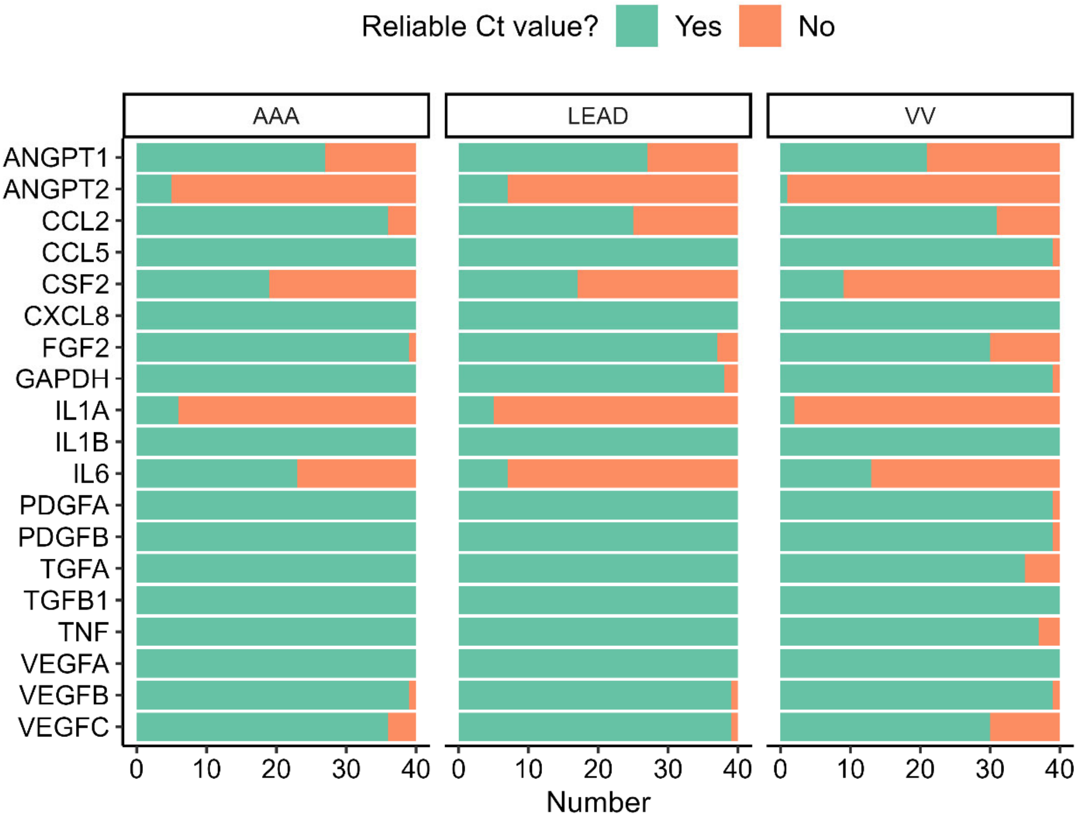

Figure S1. Numbers of samples with reliable and unreliable Ct values in gene expression dataset.

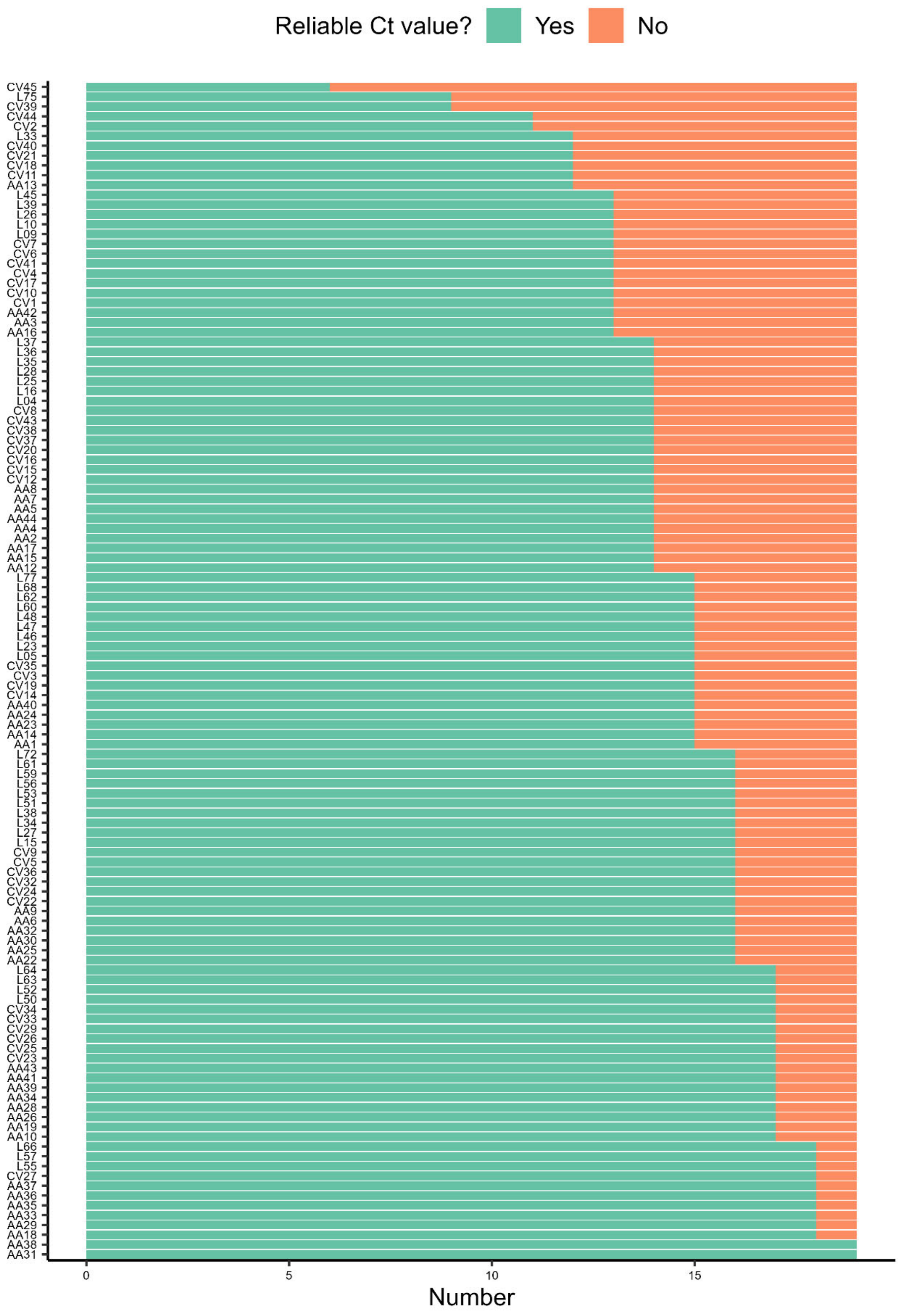

Figure S2. The amounts of genes with reliable and unreliable Ct values in gene expression dataset.

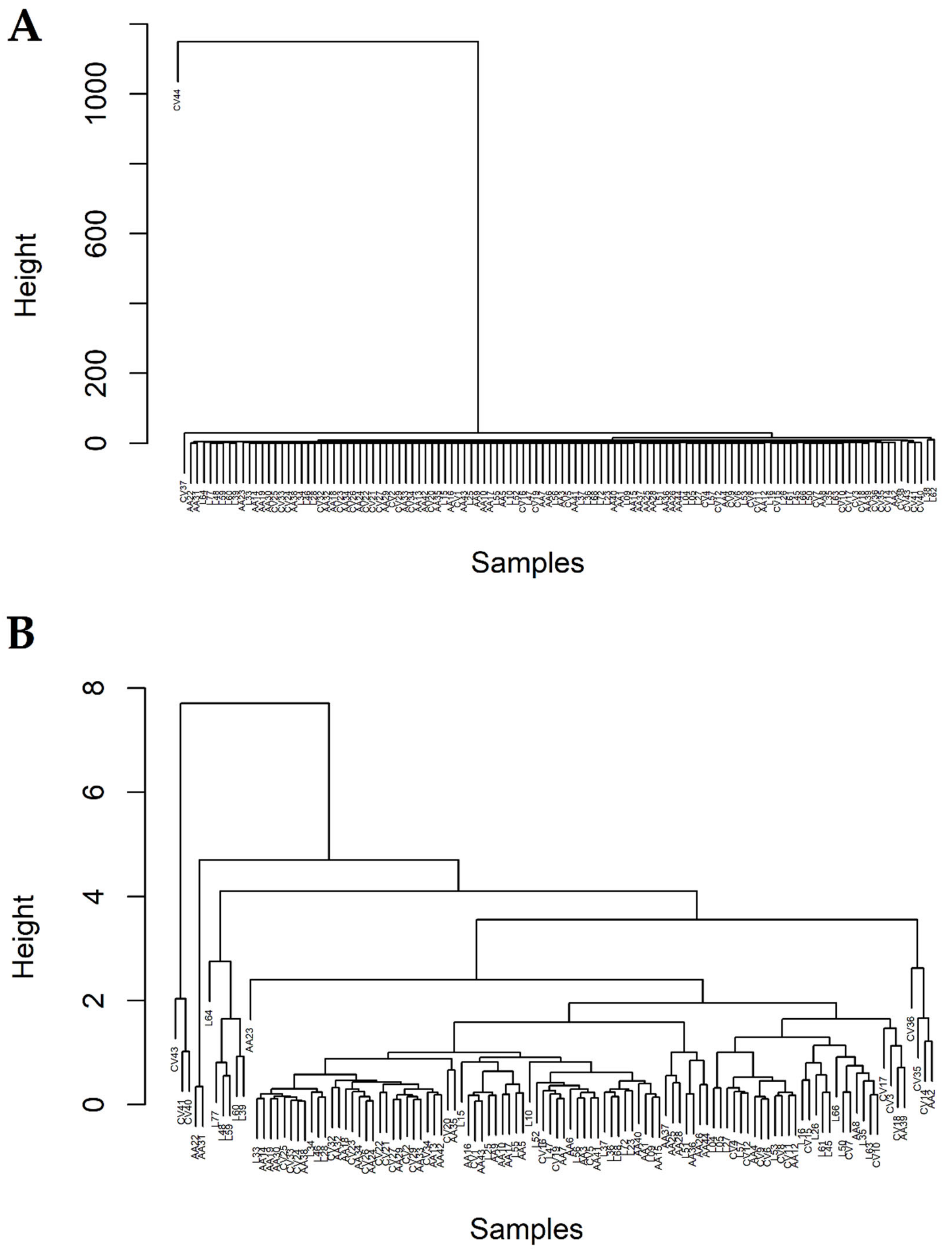

Figure S3. Hierarchical analysis performed for transformed delta Ct data in the gene expression dataset using averages of Euclidean distances, (**A**) before removing outlier samles and (**B**) after removing outlier samples.

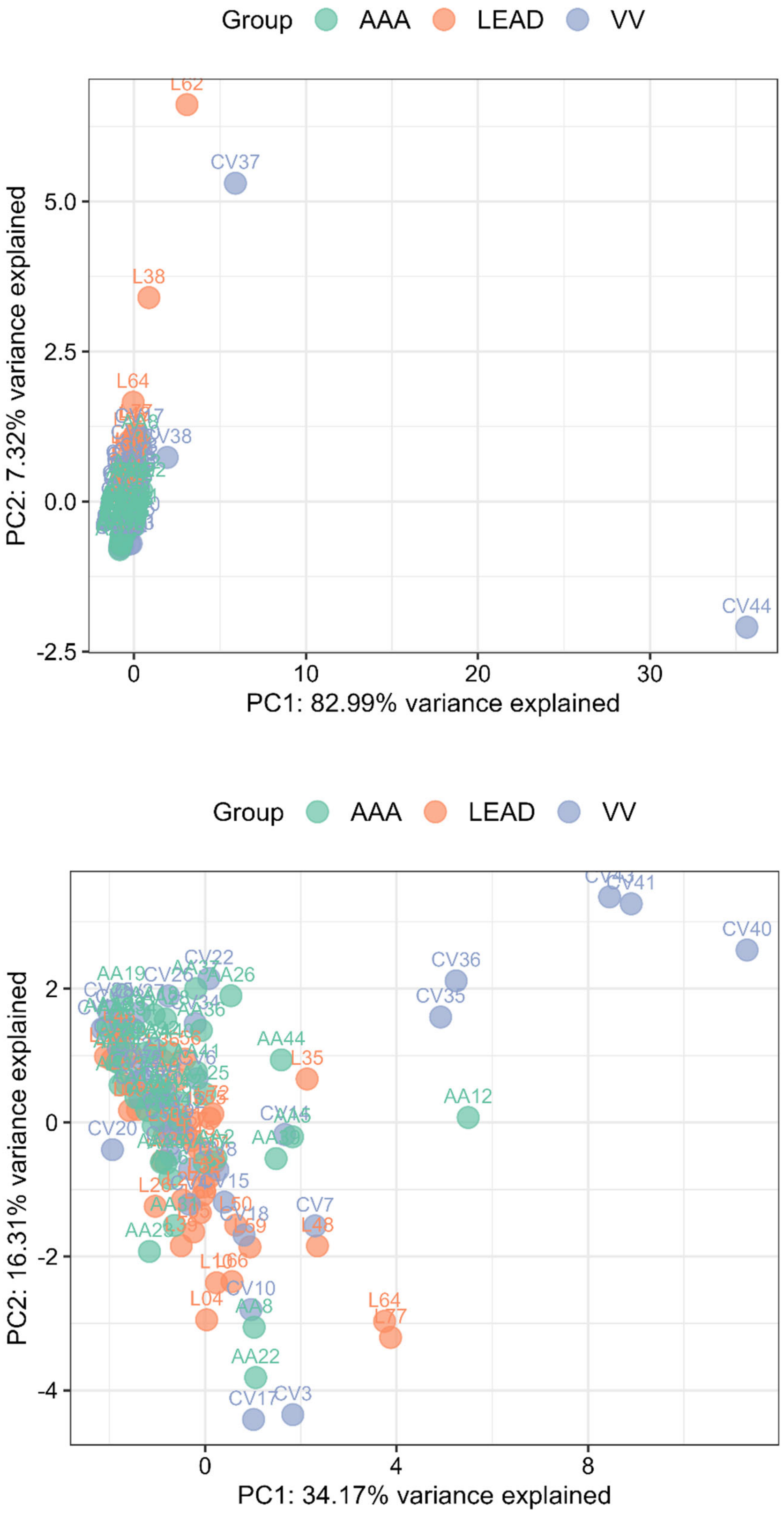

Figure S4. Spatial arrangement of samples by the first two components from principal component analysis performed for transformed delta Ct data, (**A**) before removing outlier samples and (**B**) after removing outlier samples.

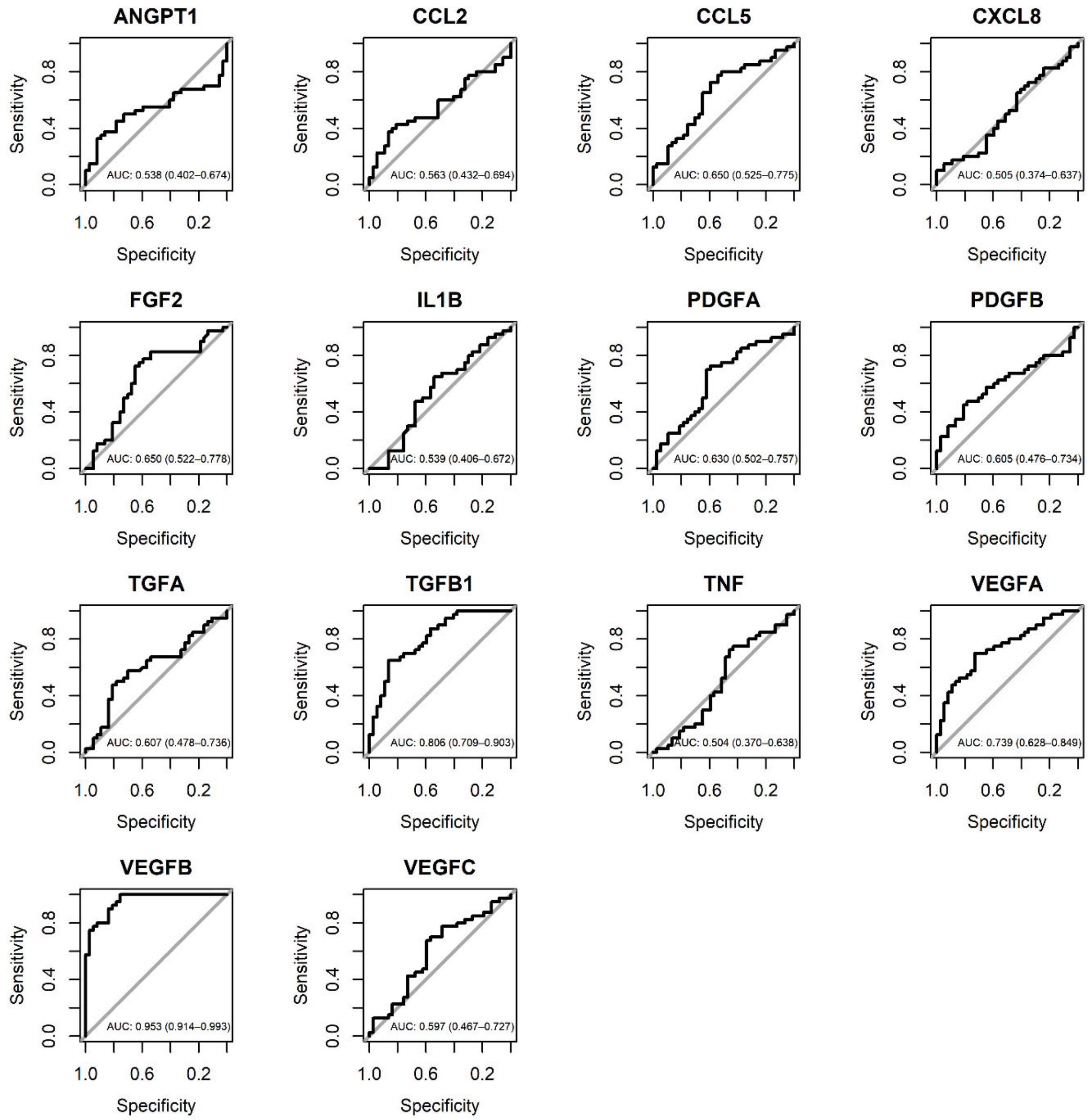

Figure S5. Results of Receiver Operating Characteristics (ROC) analysis performed for the studied genes between LEAD and AAA groups.

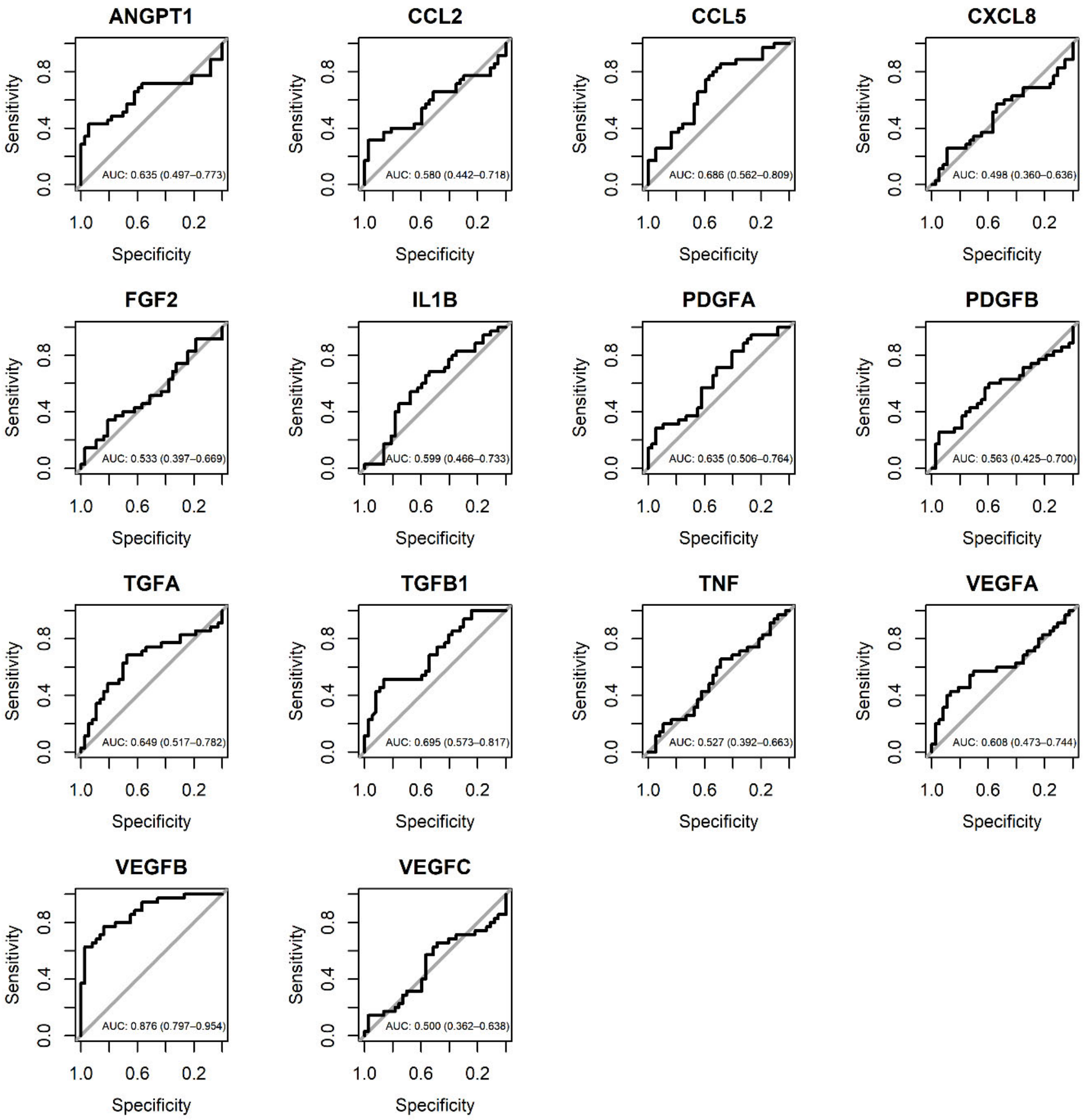

Figure S6. Results of Receiver Operating Characteristics (ROC) analysis performed for the studied genes between LEAD and VV groups.

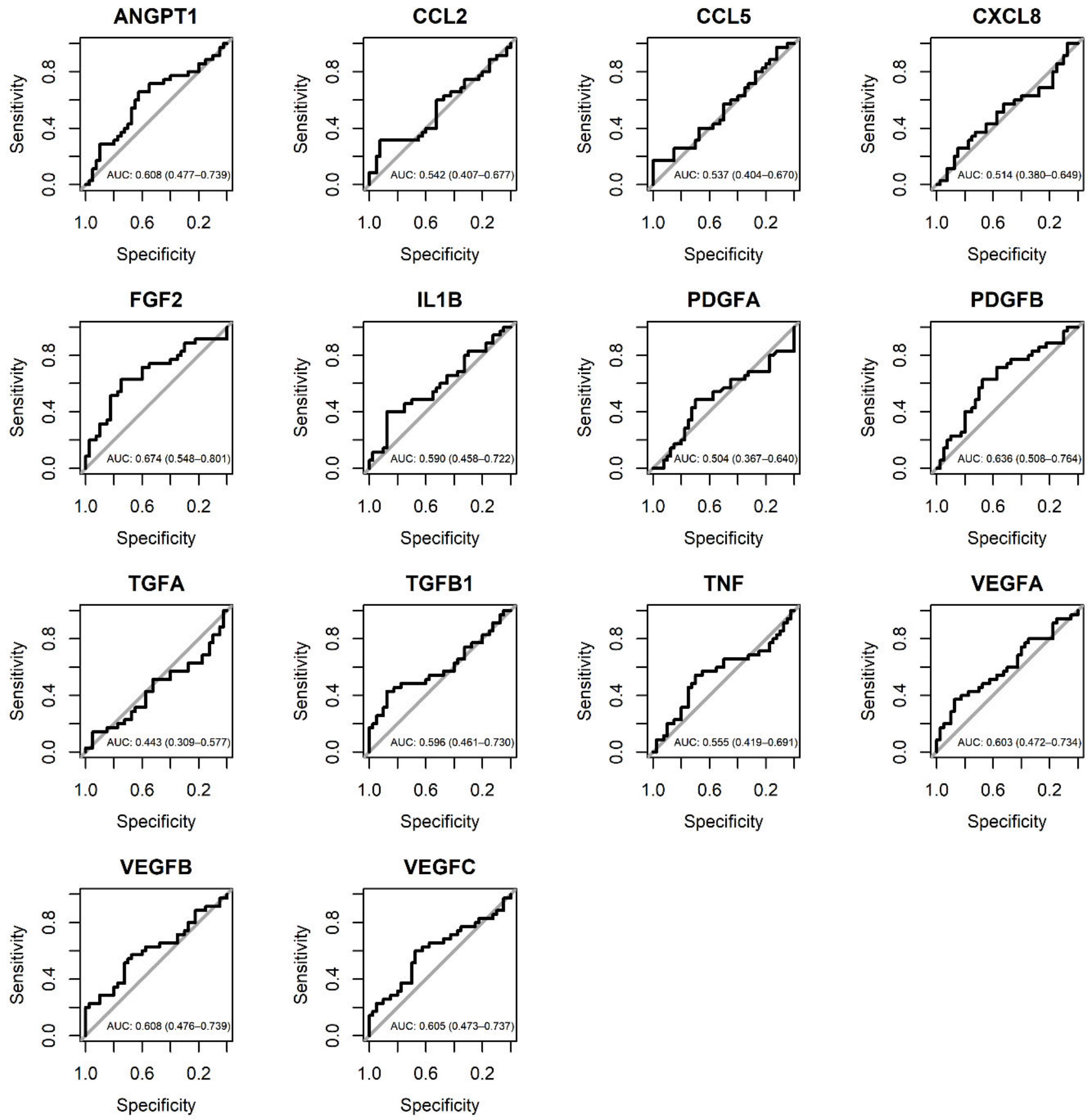

Figure S7. Results of Receiver Operating Characteristics (ROC) analysis performed for the studied genes between AAA and VV groups.

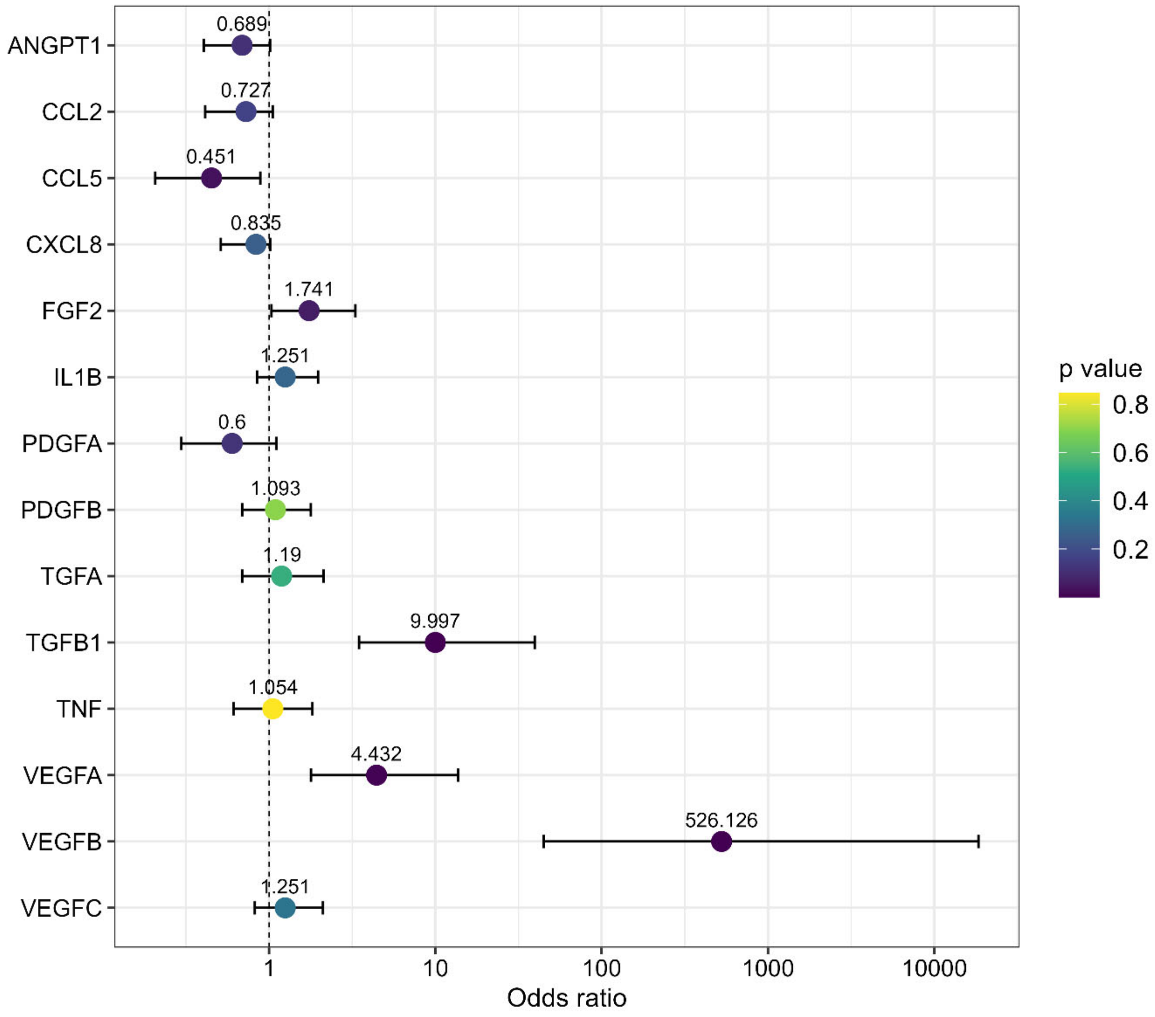

Figure S8. Odds ratios, their 95% confidence intervals, and p values obtained in the univariate logistic regression analysis for the studied genes between the LEAD and AAA groups.

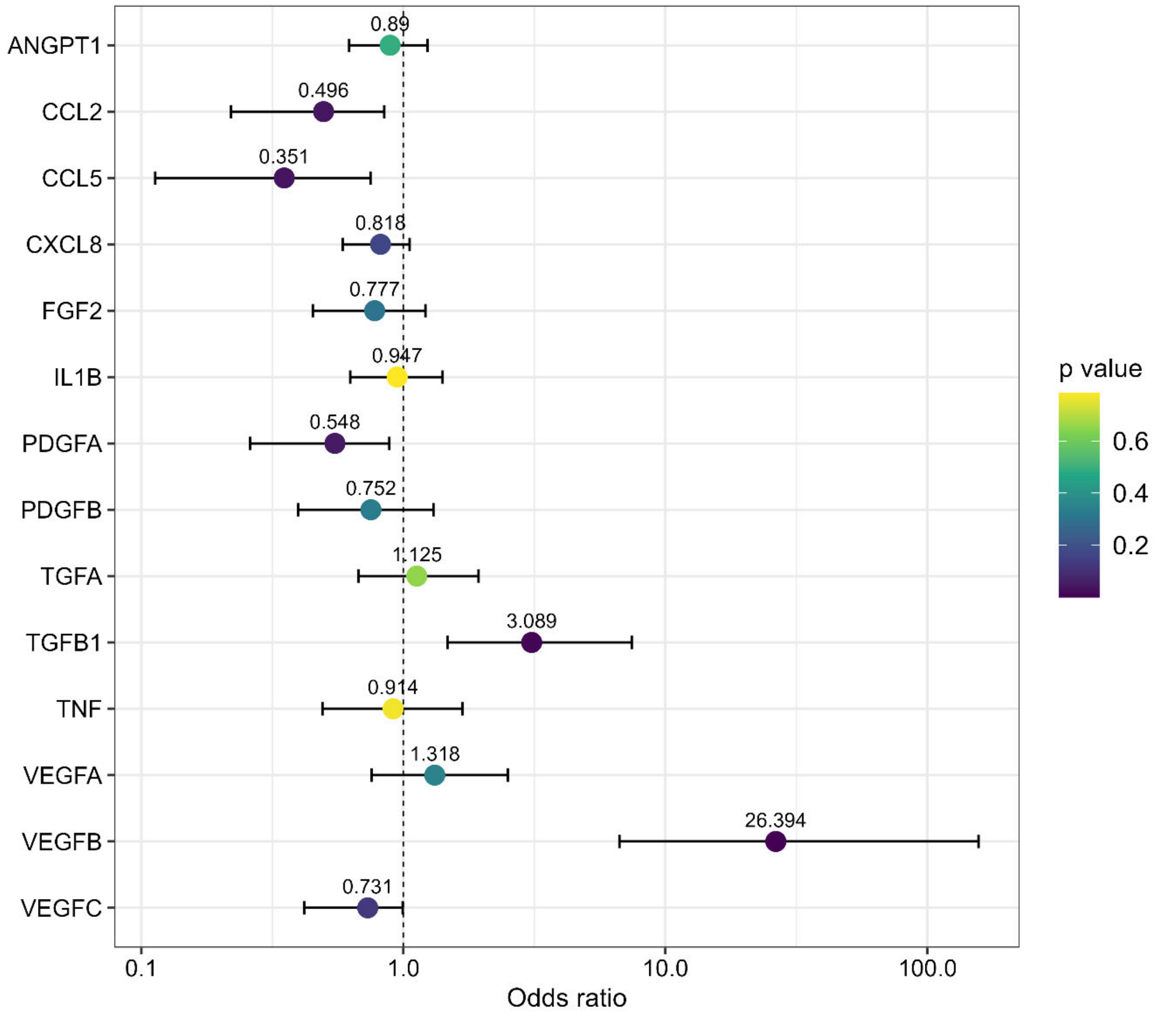

Figure S9. Odds ratios, their 95% confidence intervals, and *p* values obtained in the univariate logistic regression analysis for the studied genes between the LEAD and VV groups.

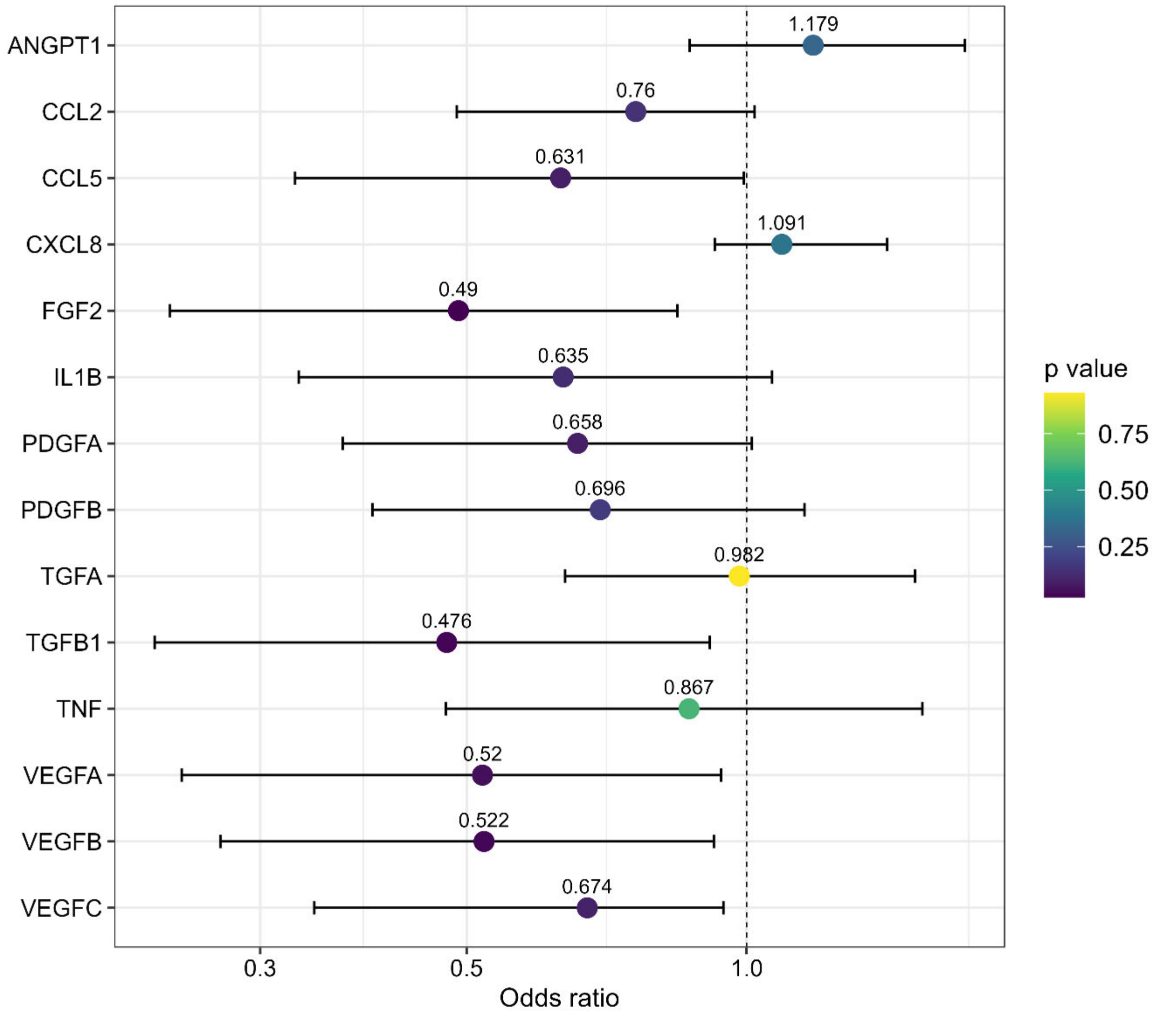

Figure S10. Odds ratios, their 95% confidence intervals, and *p* values obtained in the univariate logistic regression analysis for the studied genes between the AAA and VV groups.

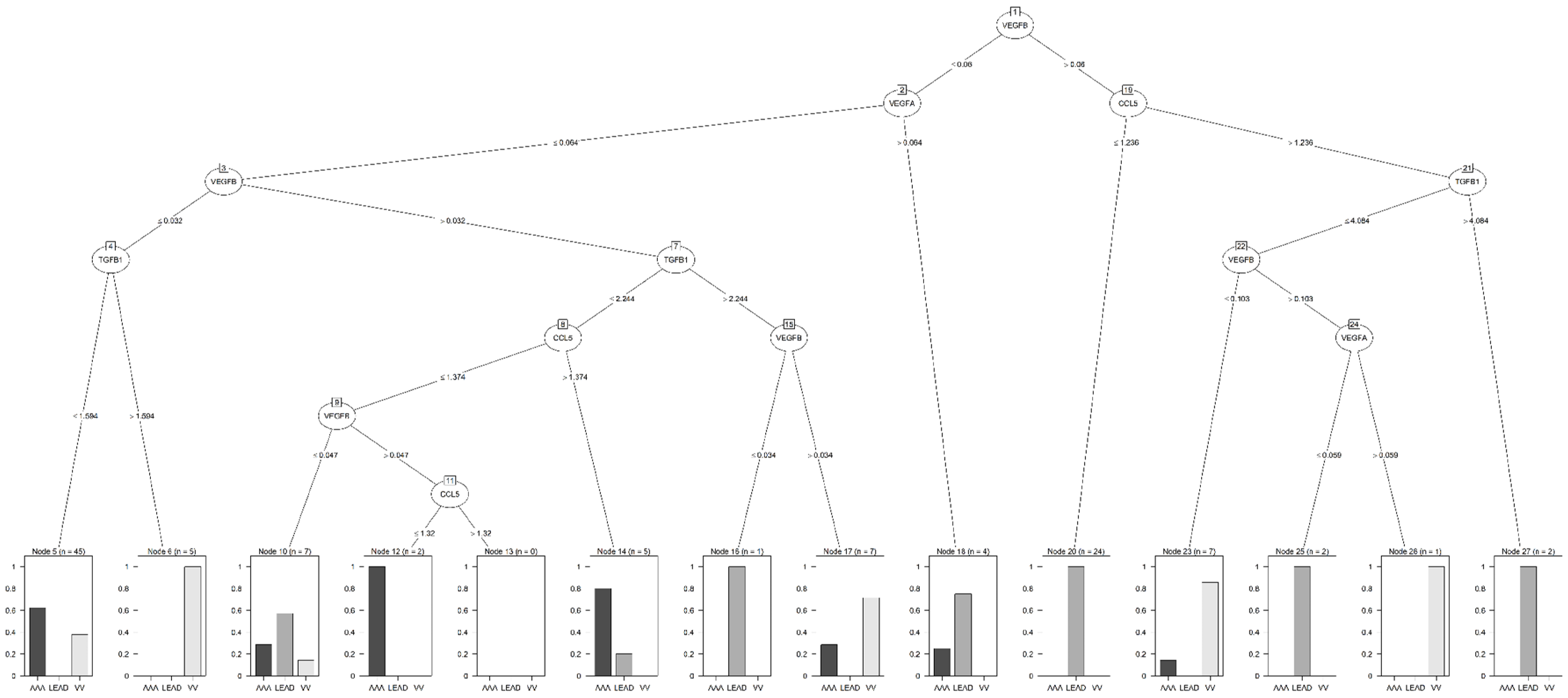

Figure S11. Decision tree generated for selected genes.

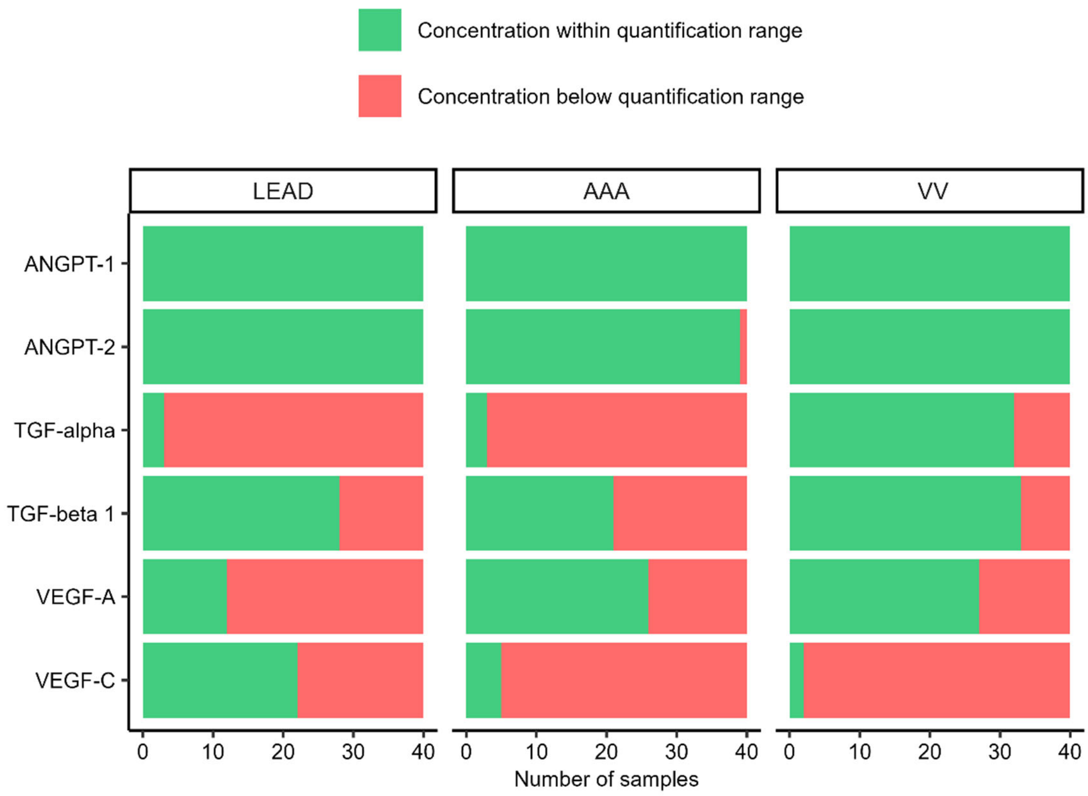

Figure S12. Amounts of samples in which proteins were quantified or not in the protein plasma level dataset.

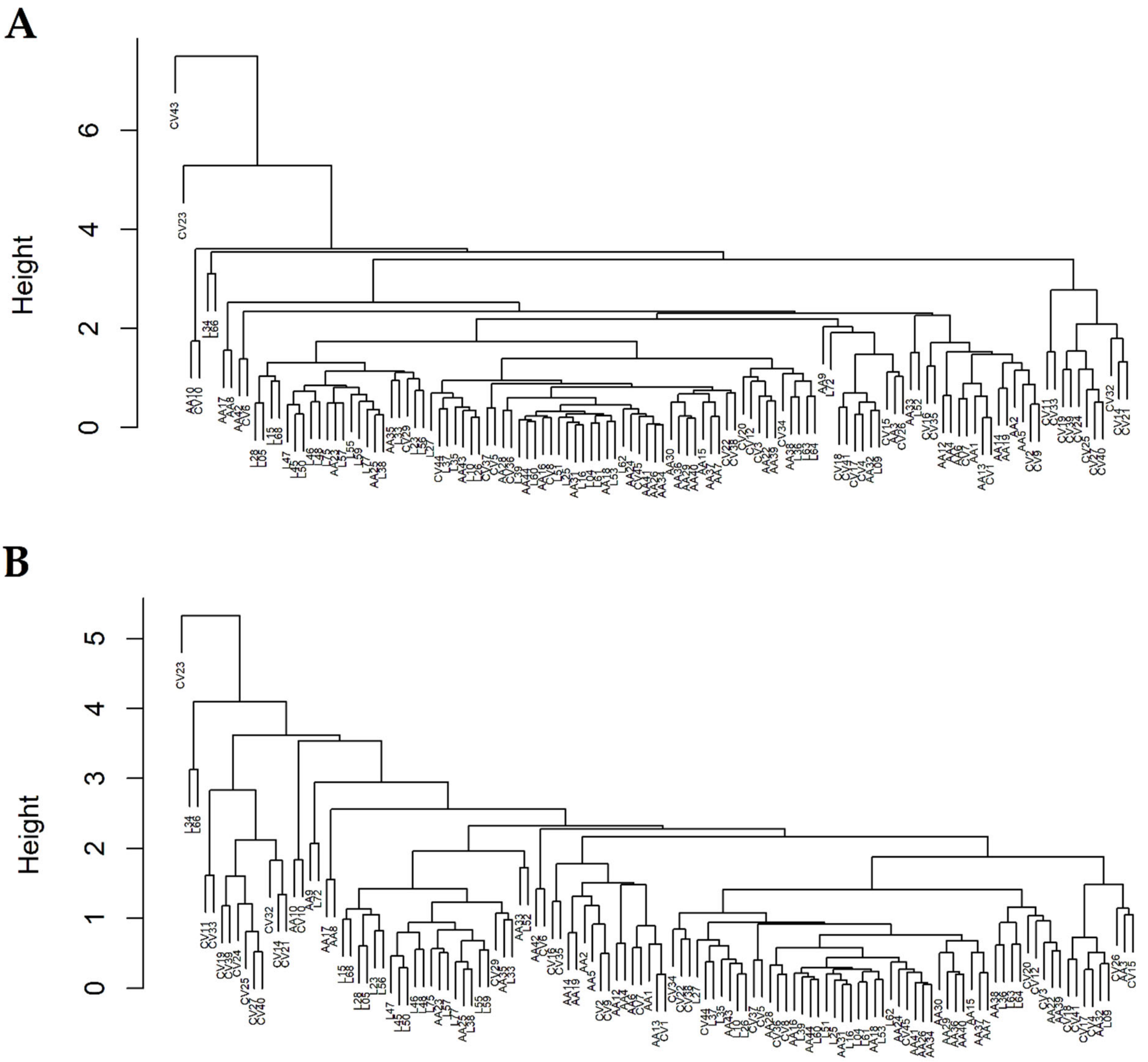

Figure S13. Hierarchical analysis performed for plasma levels of the analyzed proteins in the studied samples using Euclidean distances and the average method, (**A**) before and (**B**) after removing outlier samples.

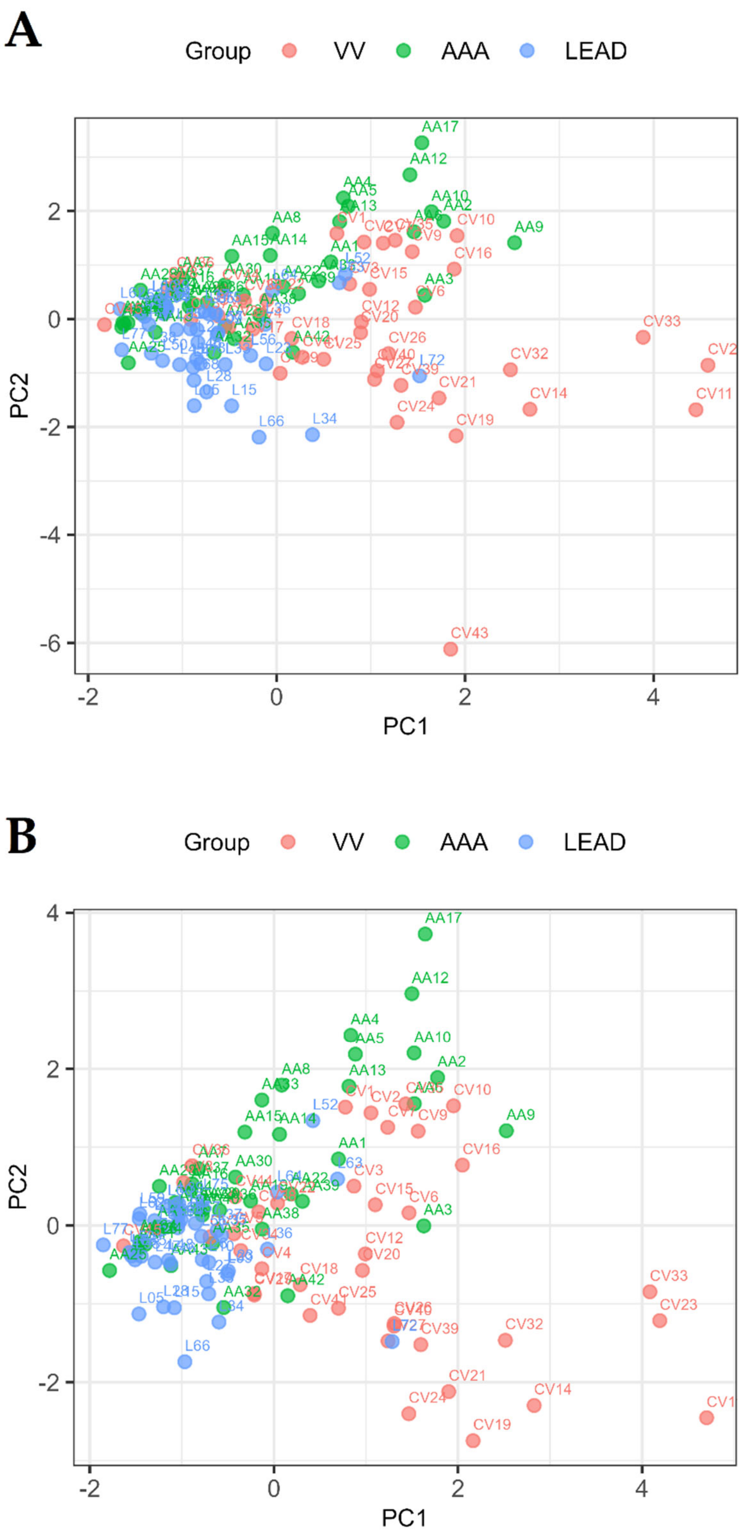

Figure S14. Spatial arrangement of samples using PCA components of protein plasma levels, (**A**) before and (**B**) after removing outlier samples.

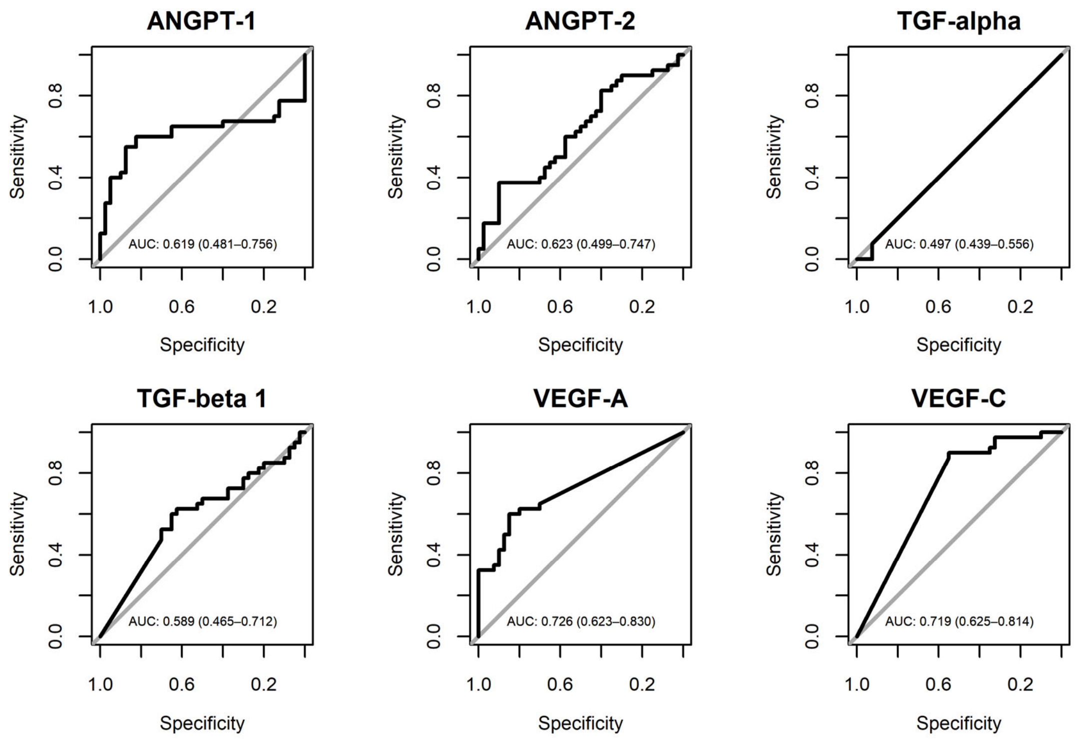

Figure S15. Results of Receiver Operating Characteristics (ROC) analysis performed for 6 analyzed proteins between the LEAD and AAA groups.

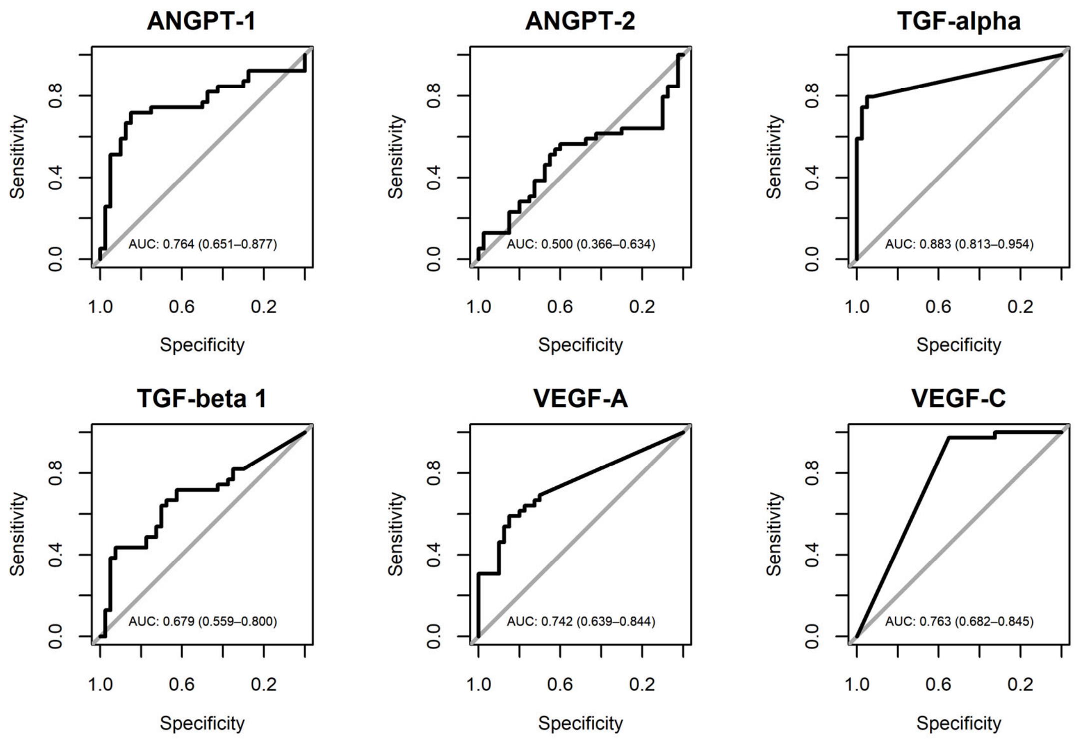

Figure S16. Results of Receiver Operating Characteristics (ROC) analysis performed for 6 analyzed proteins between the LEAD and VV groups.

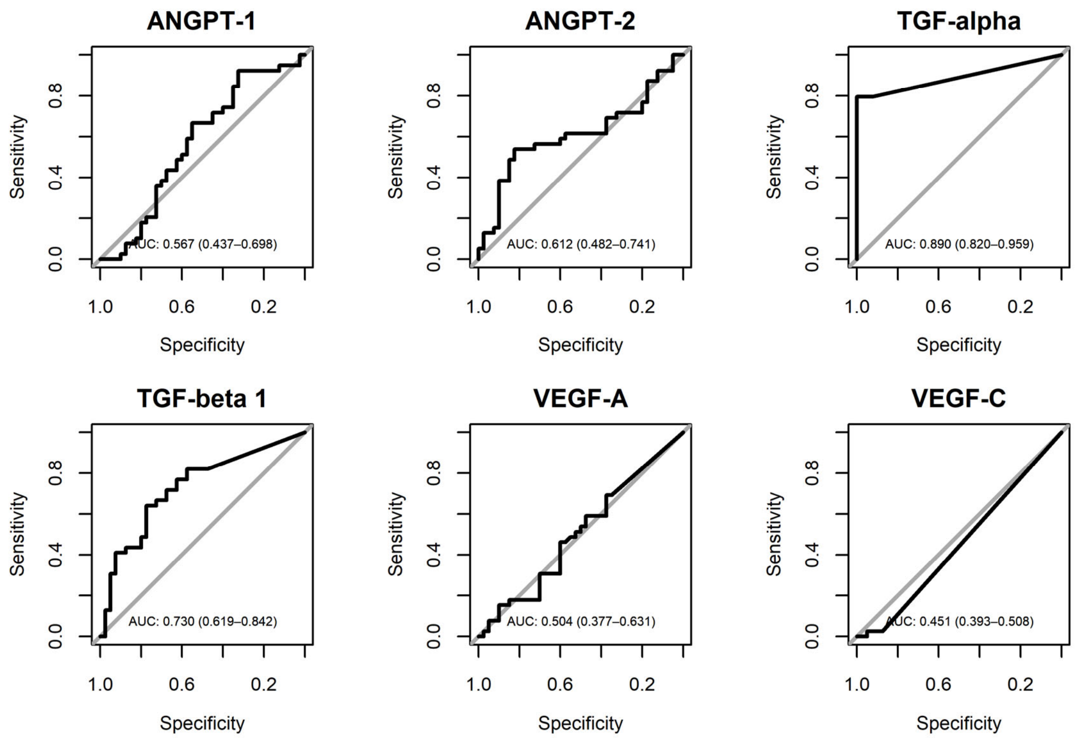

Figure S17. Results of Receiver Operating Characteristics (ROC) analysis performed for 6 analyzed proteins between AAA and VV groups.

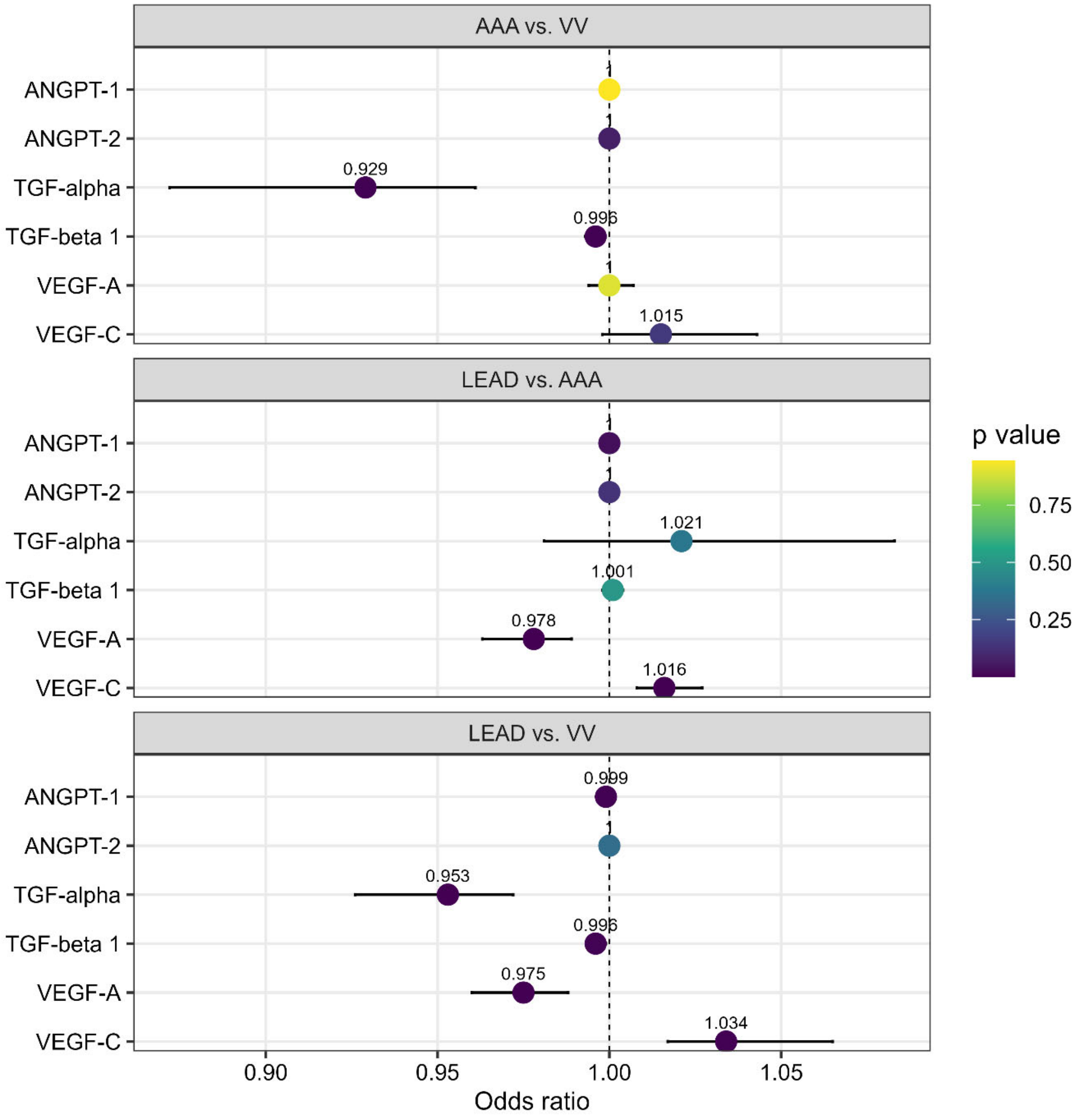

Figure S18. Odds ratios, their 95% confidence intervals, and *p* values obtained in the univariate logistic regression analysis for the studied proteins.

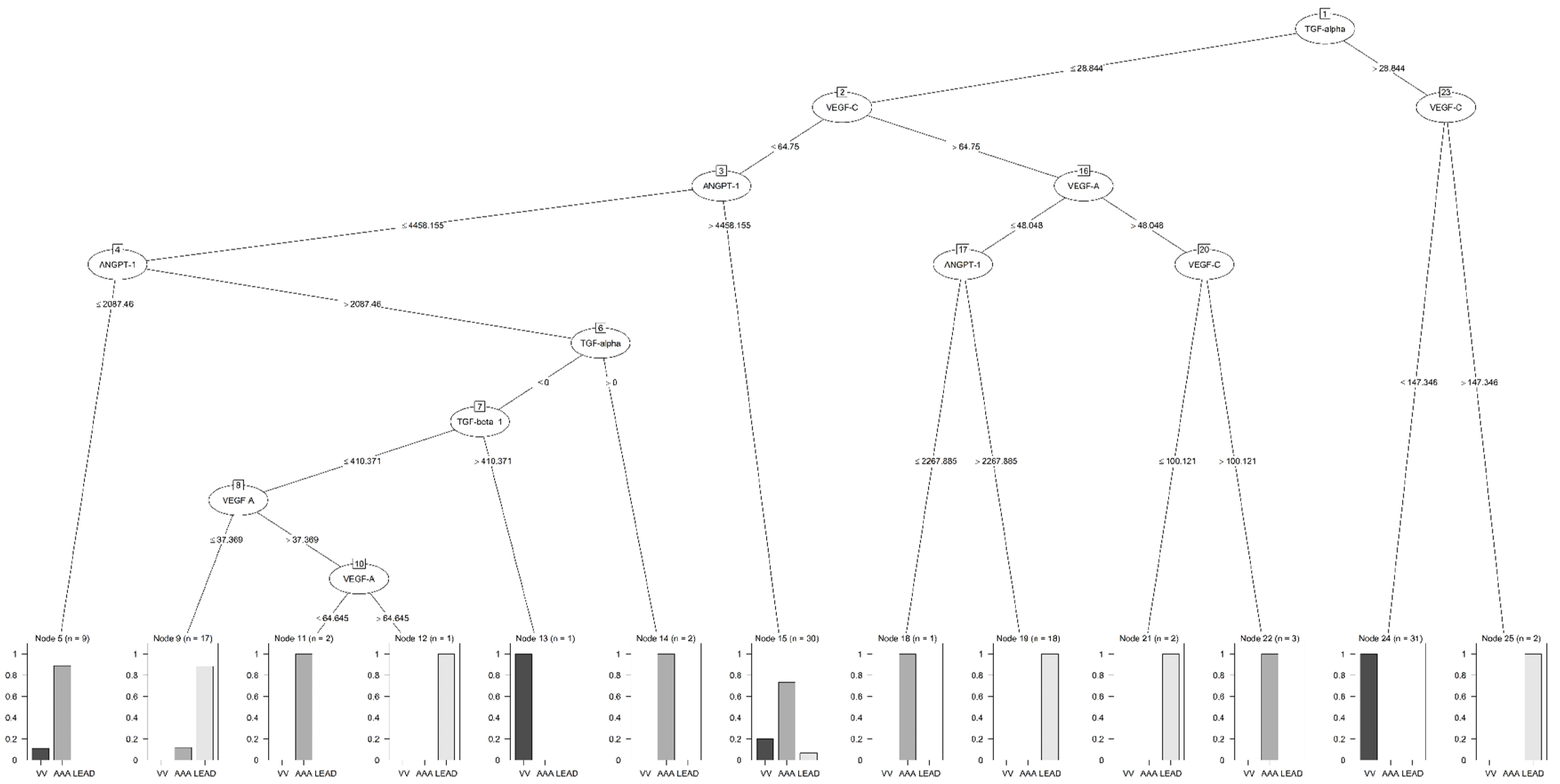

Figure S19. Decision tree generated for selected proteins.

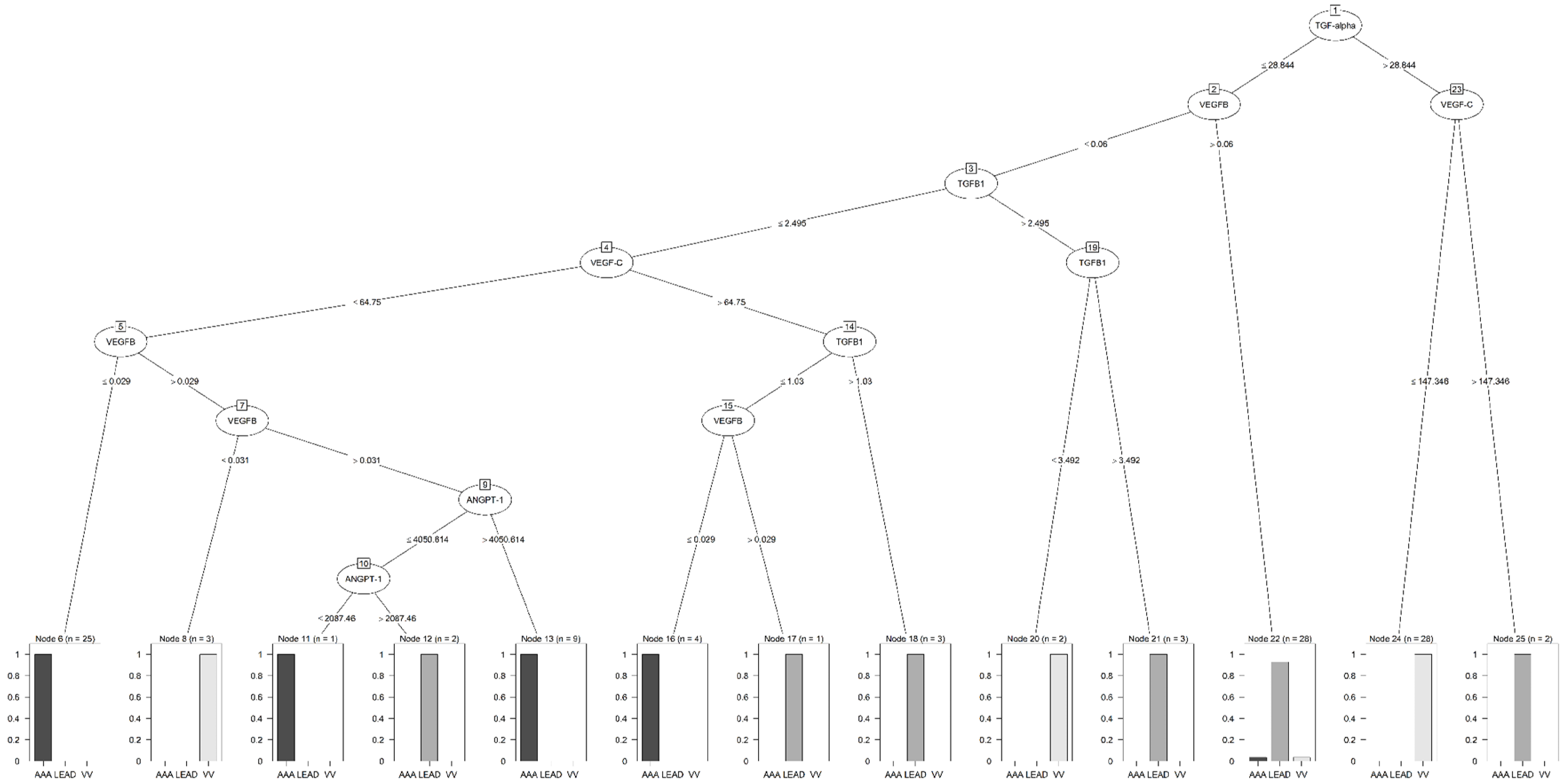

Figure S20. Decision tree generated for selected genes and proteins.

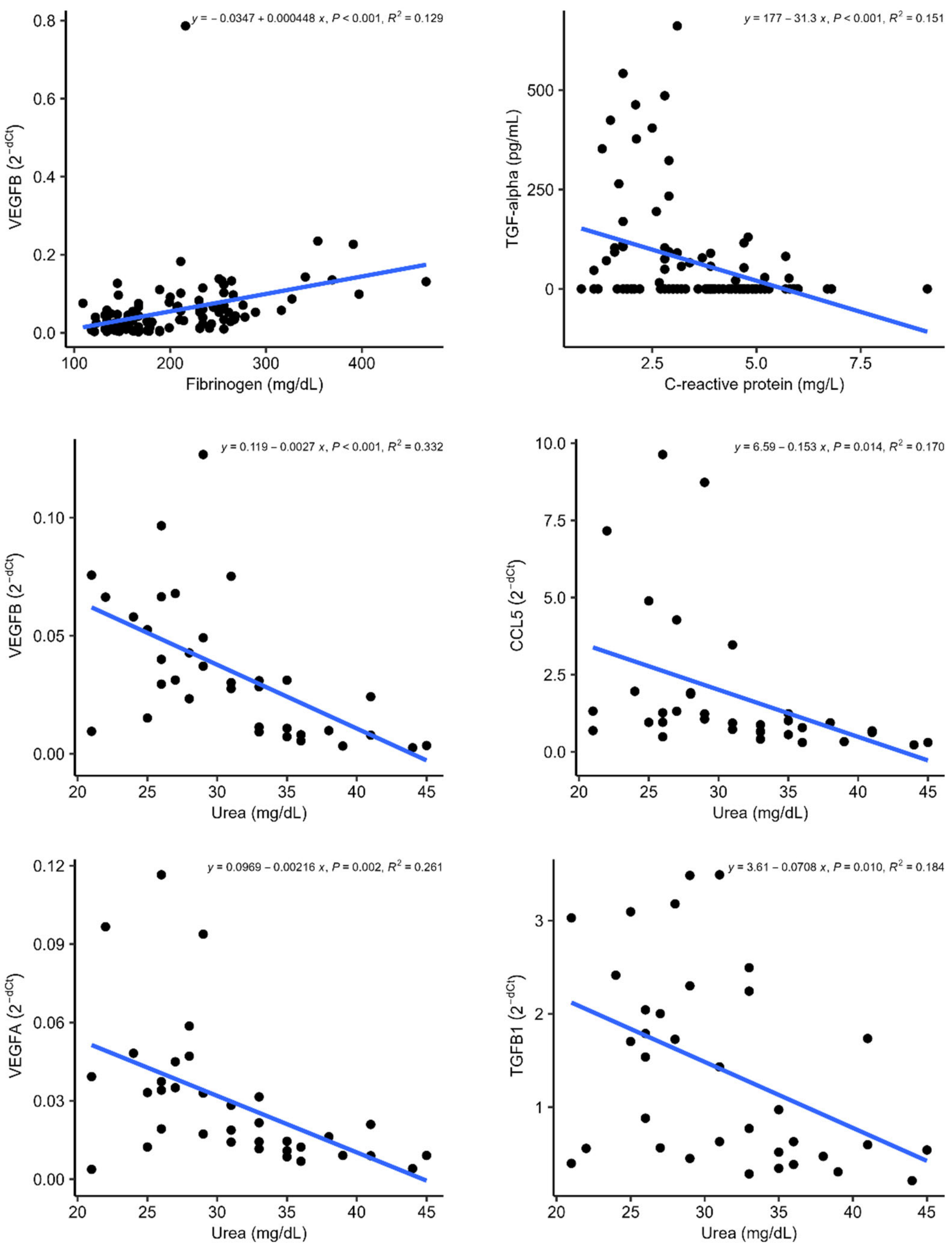

Figure S21. Statistically significant relationships between the expression of selected genes or proteins and continuous-type clinical characteristics of the study subjects.

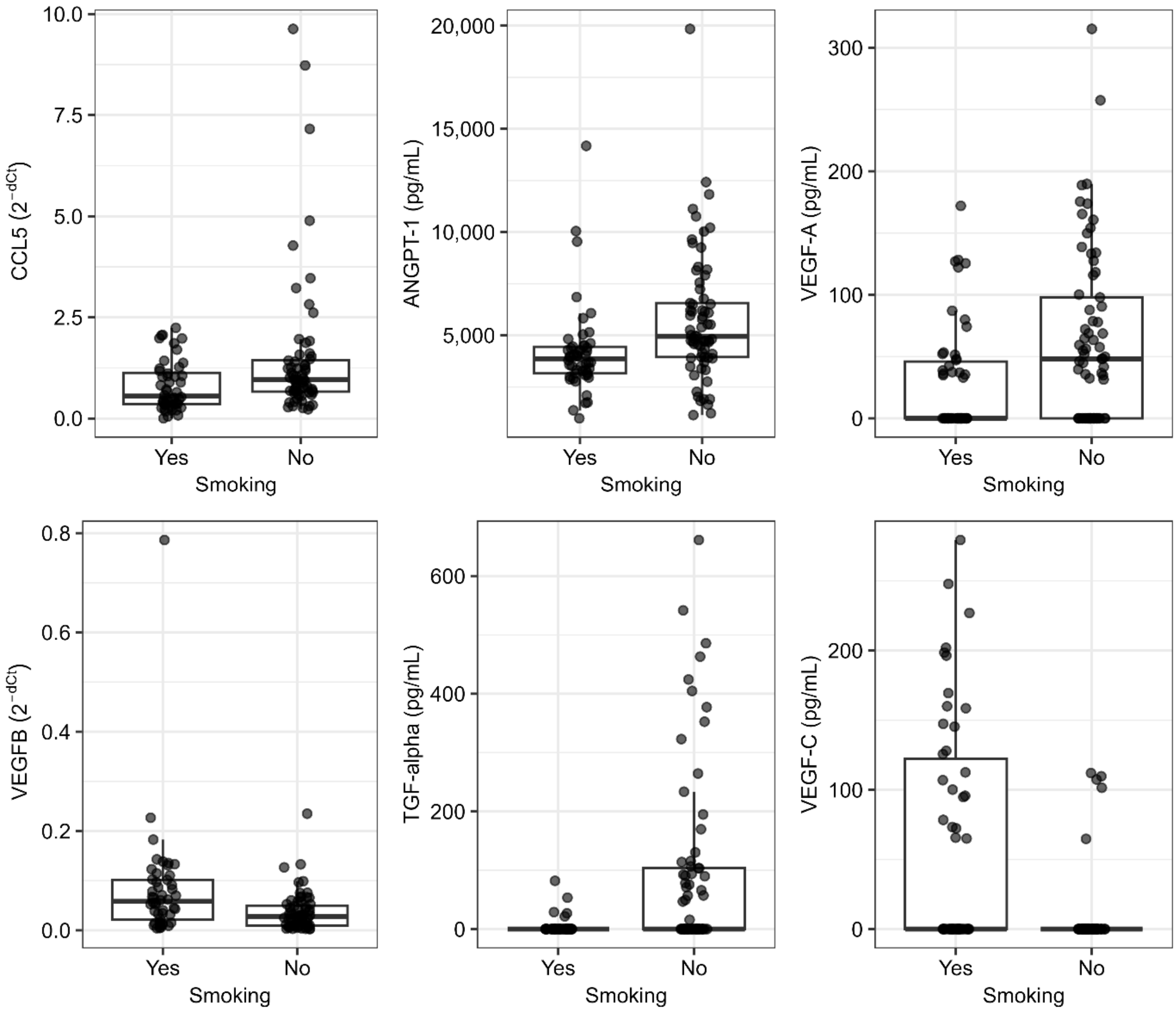

Figure S22. Distributions of expression levels of genes and plasma levels of proteins associated with smoking in the study subjects. Whiskers reach the most distant point in 1.5 interquartile range, boxes range between the 25% and 75% quartiles, and horizontal lines inside boxes mark the median value.

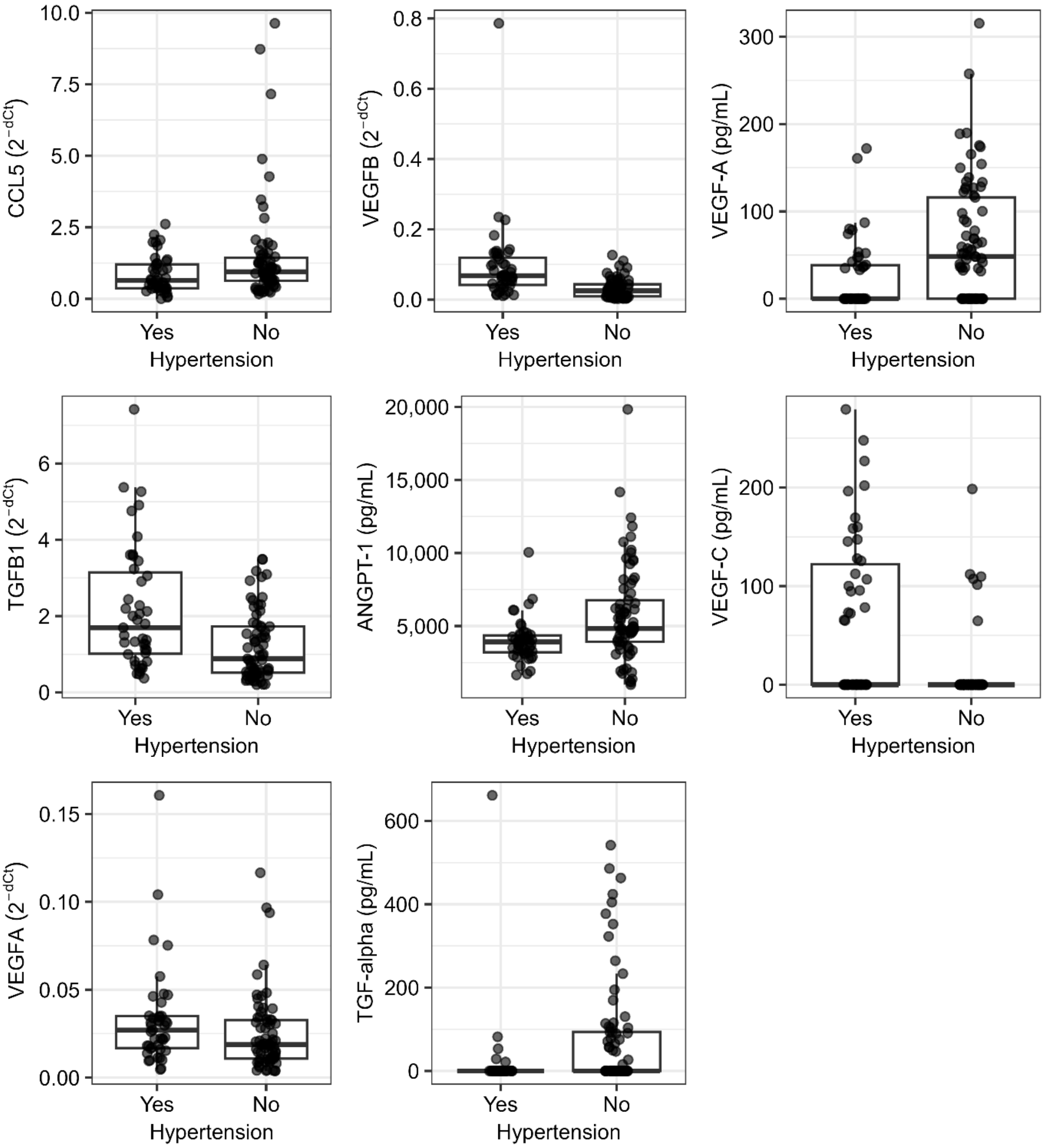

Figure S23. Distributions of expression levels of genes and plasma levels of proteins that are associated with hypertension in the study subjects. Whiskers reach the most distant point in 1.5 interquartile range, boxes range between 25% and 75% quartile, horizontal lines inside boxes mark median value.

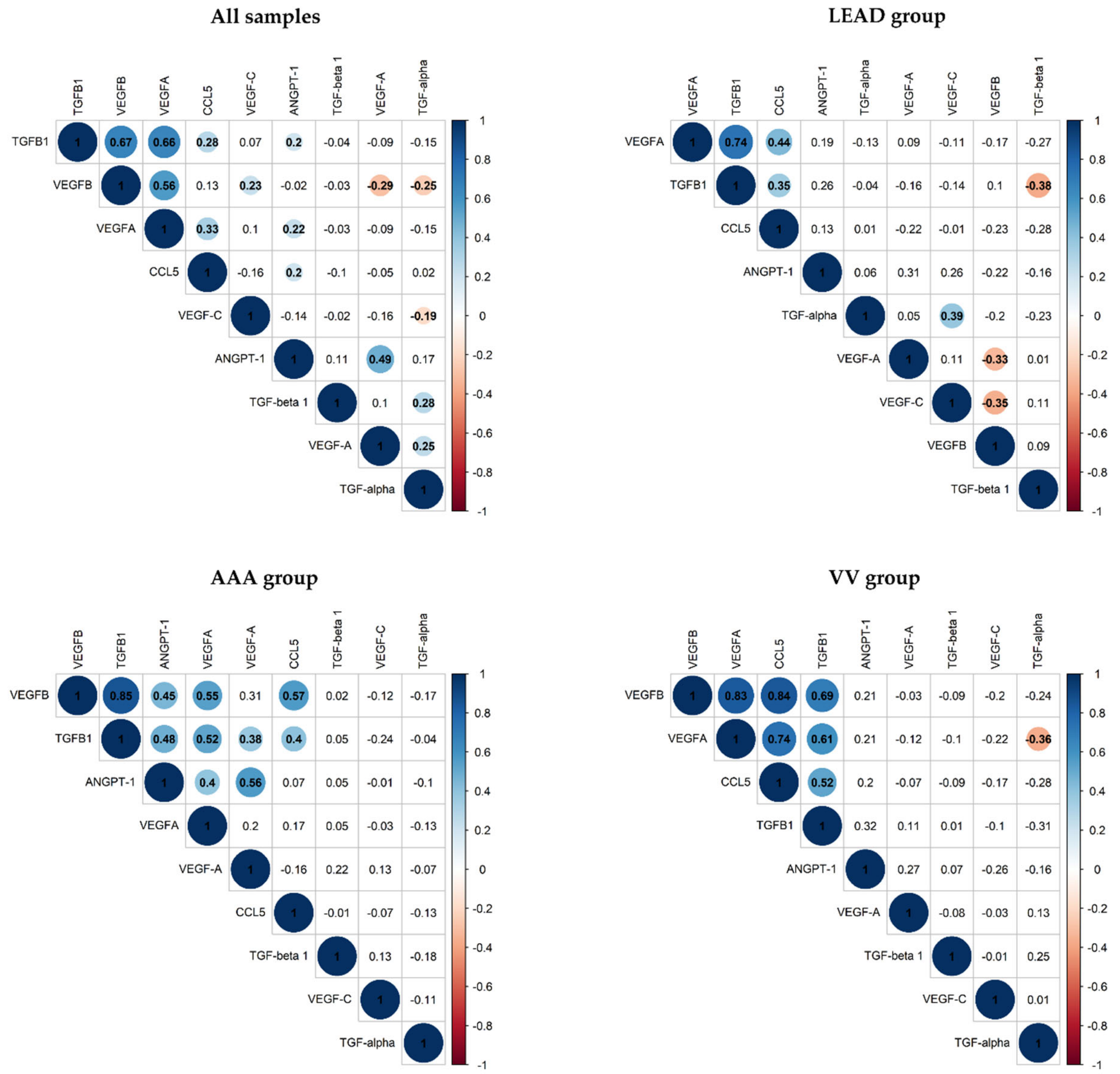

Figure S24. Correlation coefficients obtained between the expression levels of 4 selected genes (*CCL5*, *TGFB1*, *VEGFA*, and *VEGFB*) and plasma levels of 5 selected proteins (ANGPT-1, TGF-alpha, TGF-beta 1, VEGF-A, and VEGF-C), calculated using Spearman rank correlation test. The plot was generated using the corrplot 0.92 package in R. Colored circles mark the correlation coefficients with statistical significance ($p < 0.05$).

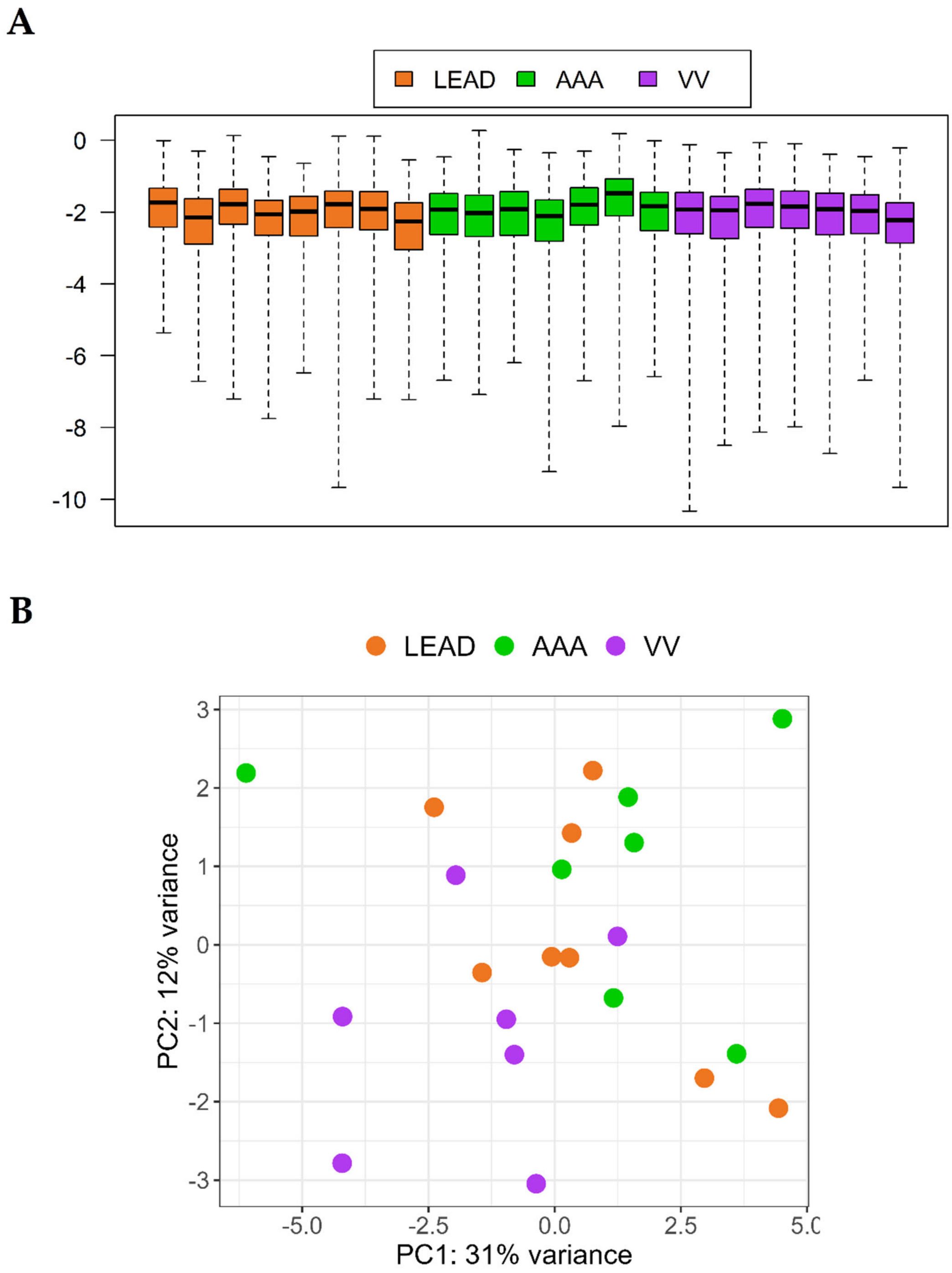

Figure S25. Quality control of RNA-seq expression data. (**A**) Boxplot presenting Cook's distances of transcription factors across samples. Whiskers define the range between the minimum and maximum values of Cook's distance; boxes range between 25% and 75% quartile; horizontal lines inside boxes mark the median value. (**B**) Spatial arrangement of the samples using PCA.

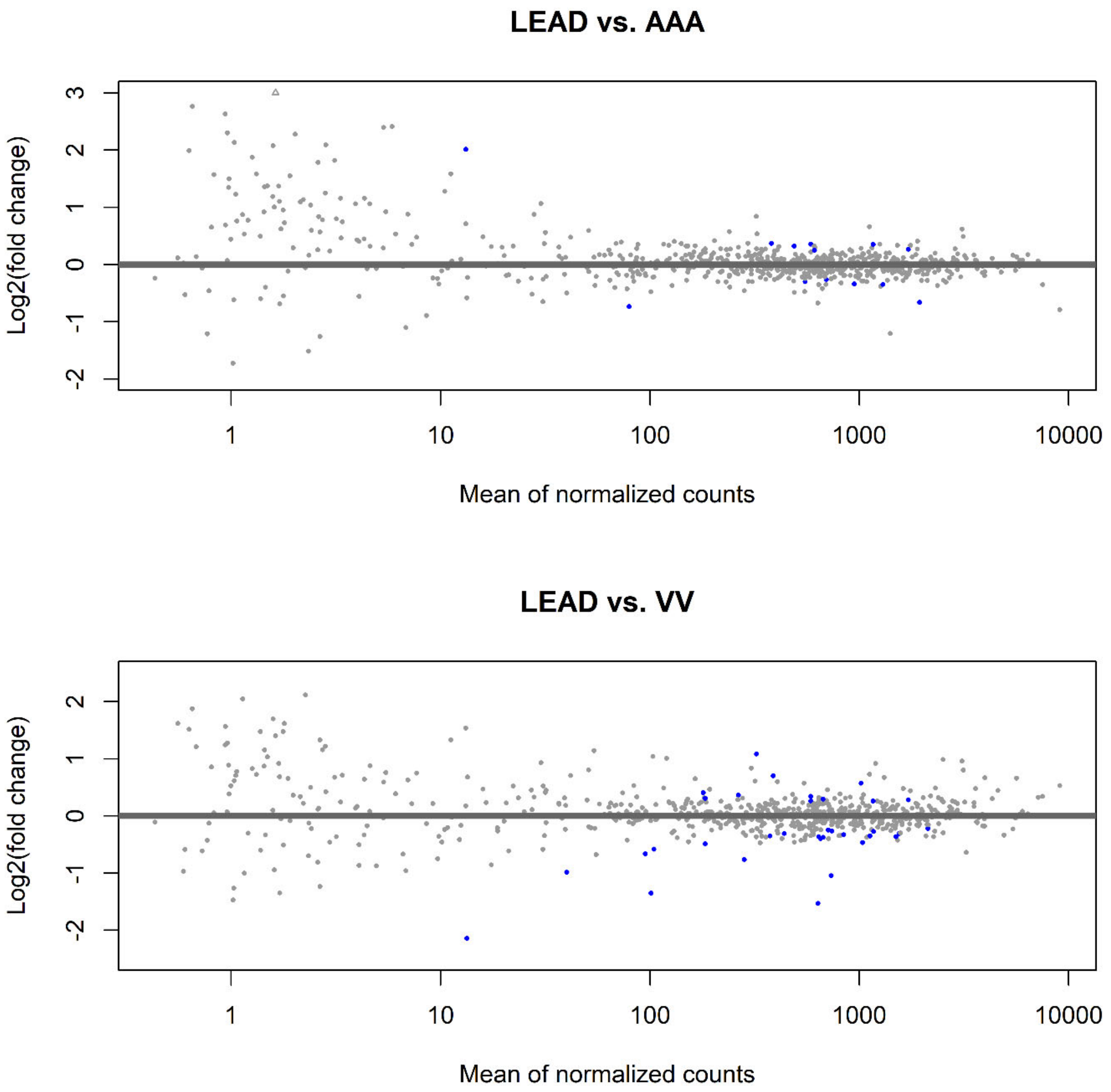

Figure S26. MA plots showing the relationship between log2 fold changes in differentially expressed transcription factors and averages of their normalized counts for comparisons. Transcription factors with FDR < 0.05 were marked as blue points.

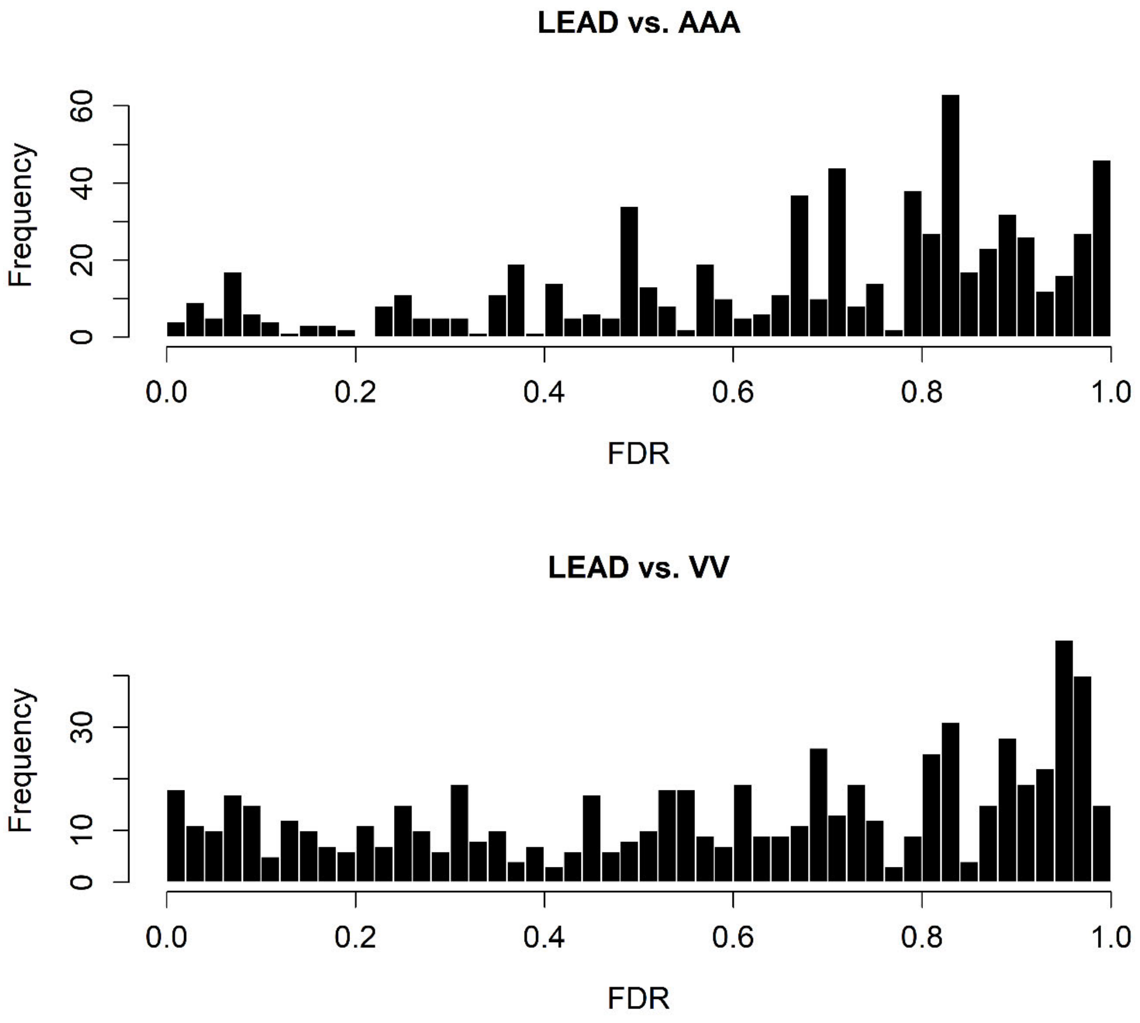

Figure S27. Histograms showing the distribution of FDR values obtained from comparisons performed using the DESeq2 package.

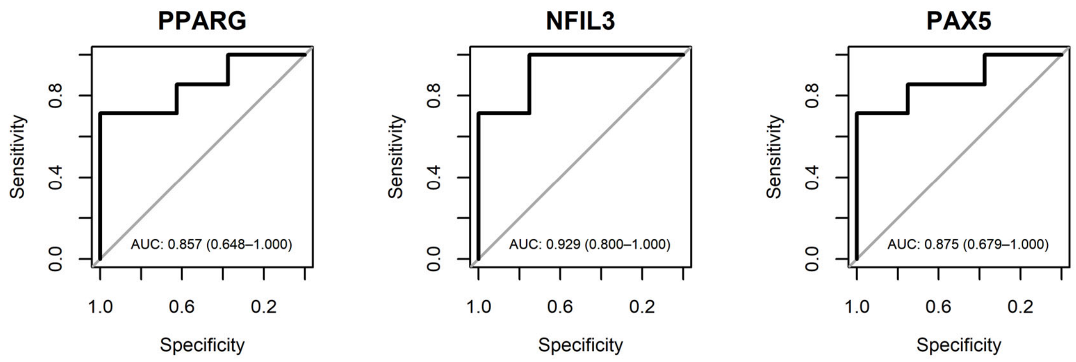

Figure S28. Results of Receiver Operating Characteristics (ROC) analysis performed for three selected transcription factors.

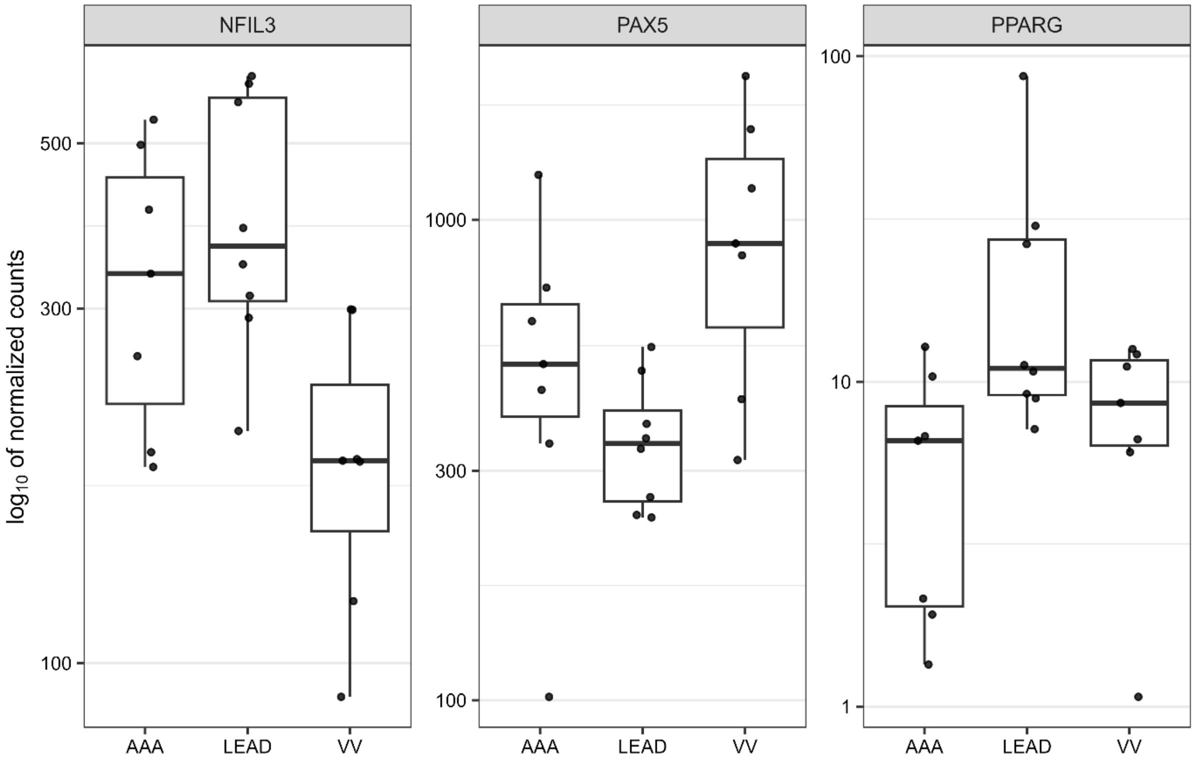

Figure S29. Distribution of expression values for three selected transcription factors in the LEAD, AAA, and VV groups. Whiskers reach the most distant point in the doubled interquartile range. Boxes range between 25% and 75% quartiles. Horizontal lines inside boxes mark the median values.

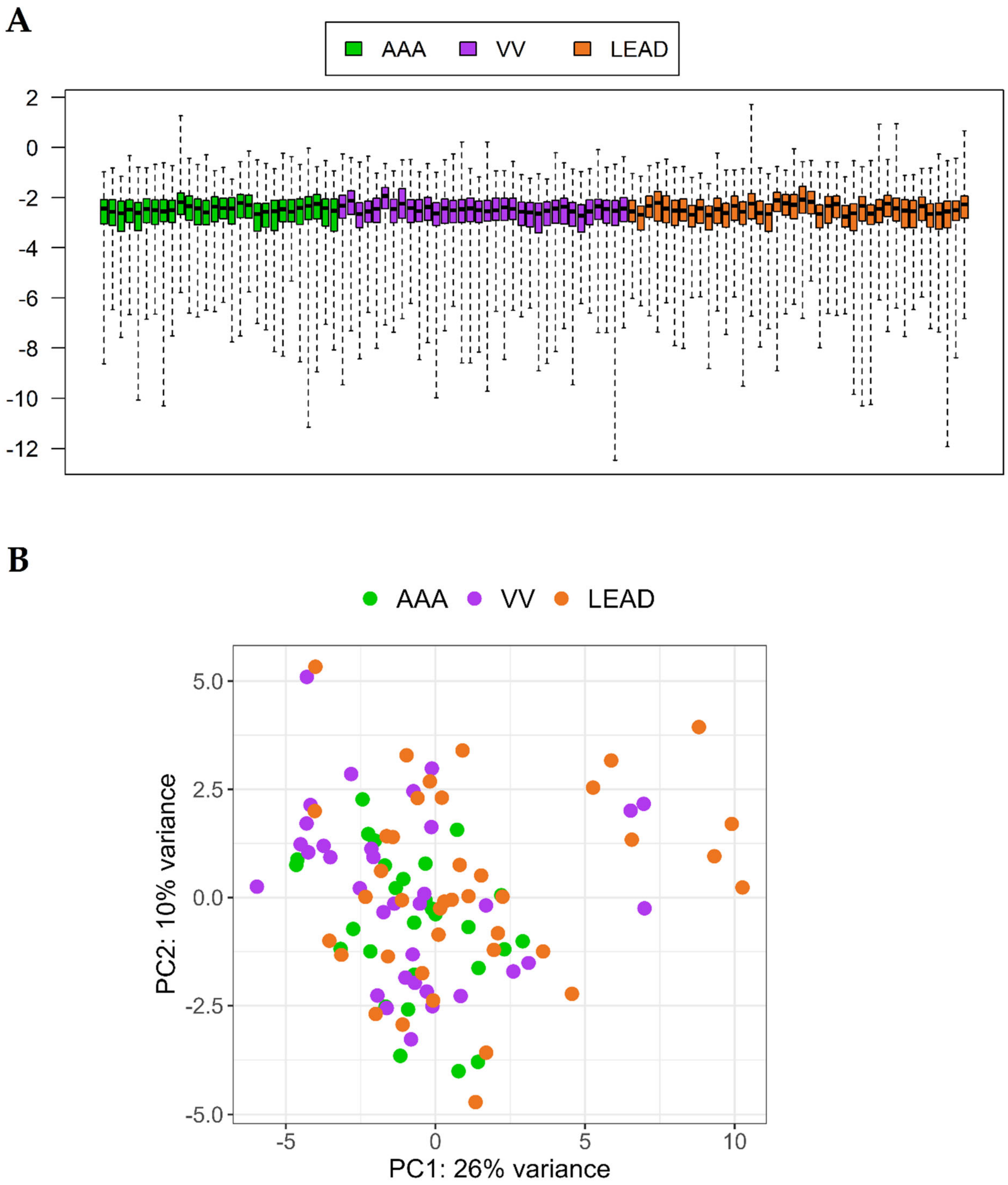

Figure S30. Quality control of miRNA expression data. (**A**) Boxplot presenting Cook's distances of transcription factors across samples. Whiskers define the range between the minimum and maximum values of Cook's distance; boxes range between 25% and 75% quartile; horizontal lines inside boxes mark the median value. (**B**) Spatial arrangement of the samples using PCA.

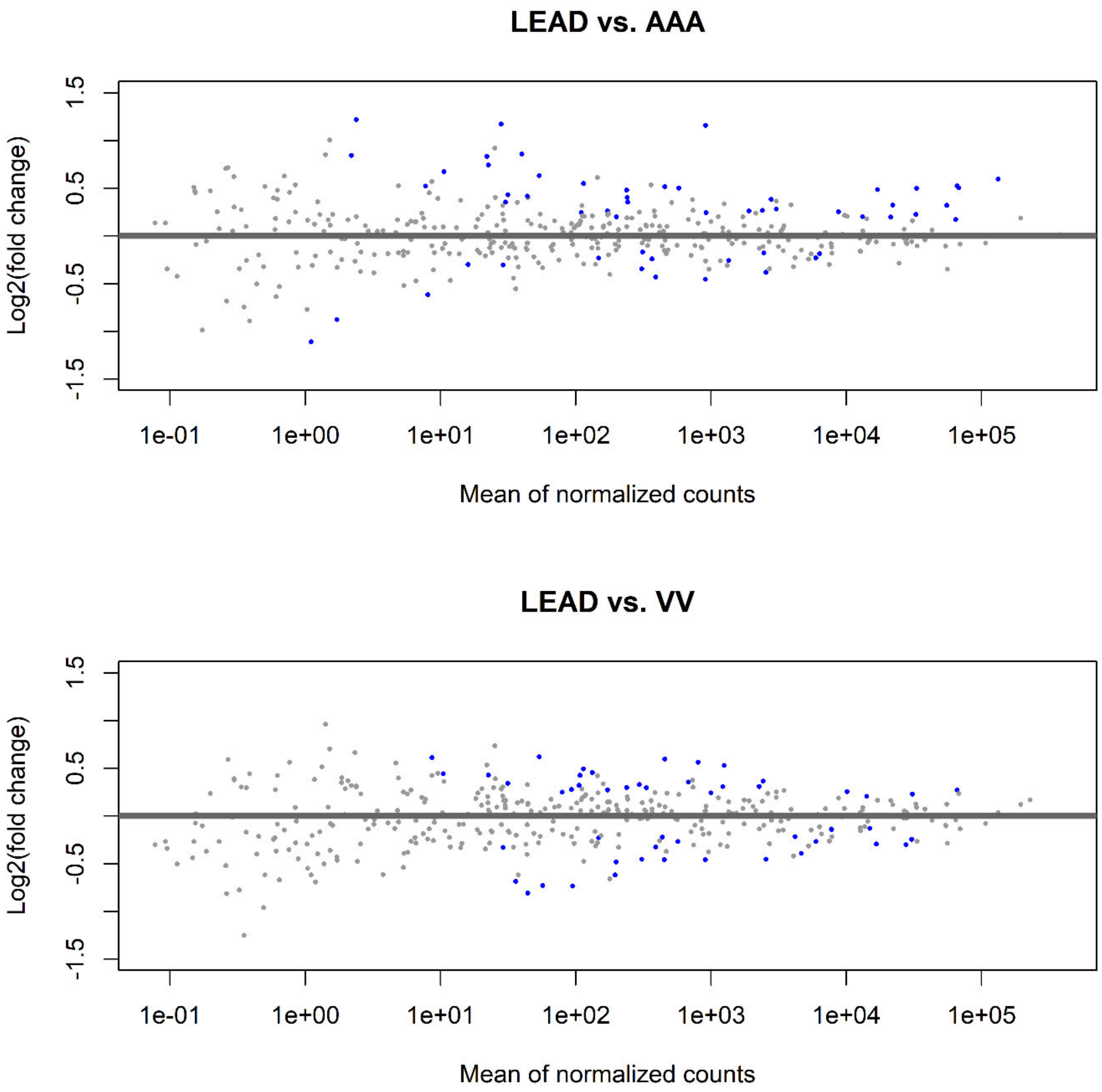

Figure S31. MA plots showing the relationship between log2 fold changes in differentially expressed miRNAs and averages of their normalized counts for comparisons. Transcription factors with FDR < 0.05 were marked as blue points.

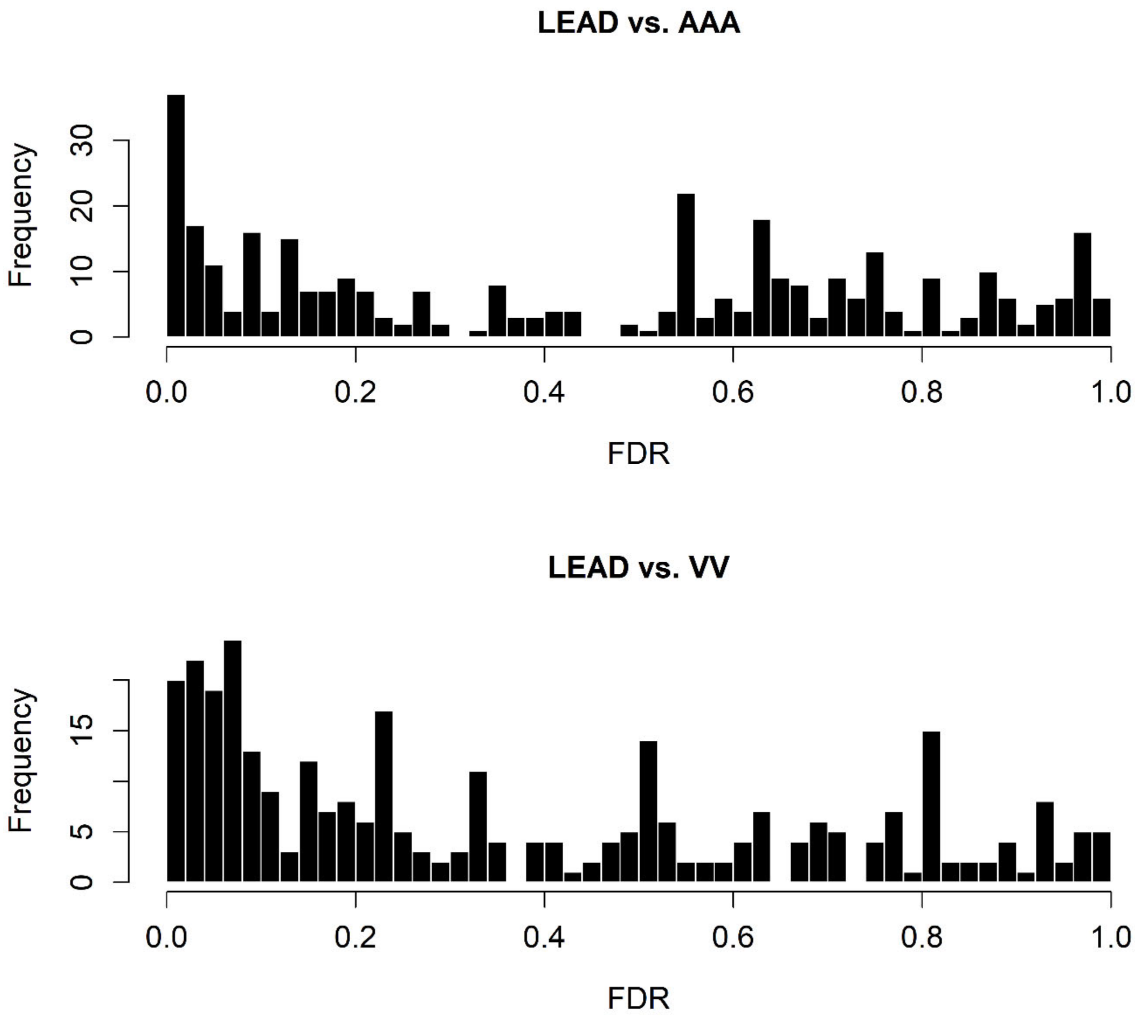

Figure S32. Histograms showing the distribution of FDR values obtained from comparisons performed using the DESeq2 package.

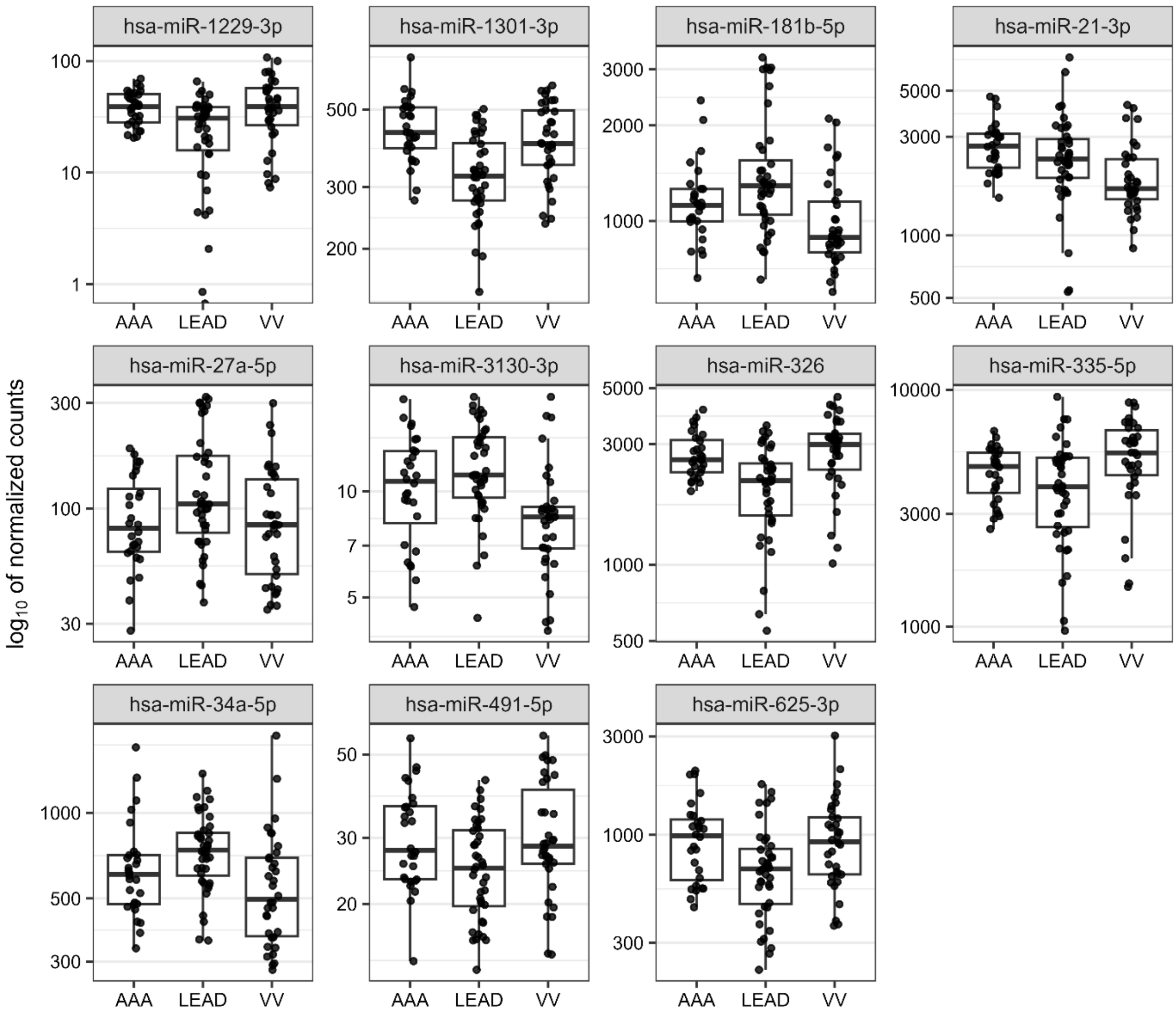

Figure S33. Distribution of expression values for selected miRNAs in the LEAD, AAA, and VV groups. Whiskers reach the most distant point in the doubled interquartile range. Boxes range between 25% and 75% quartiles. Horizontal lines inside boxes mark the median values.

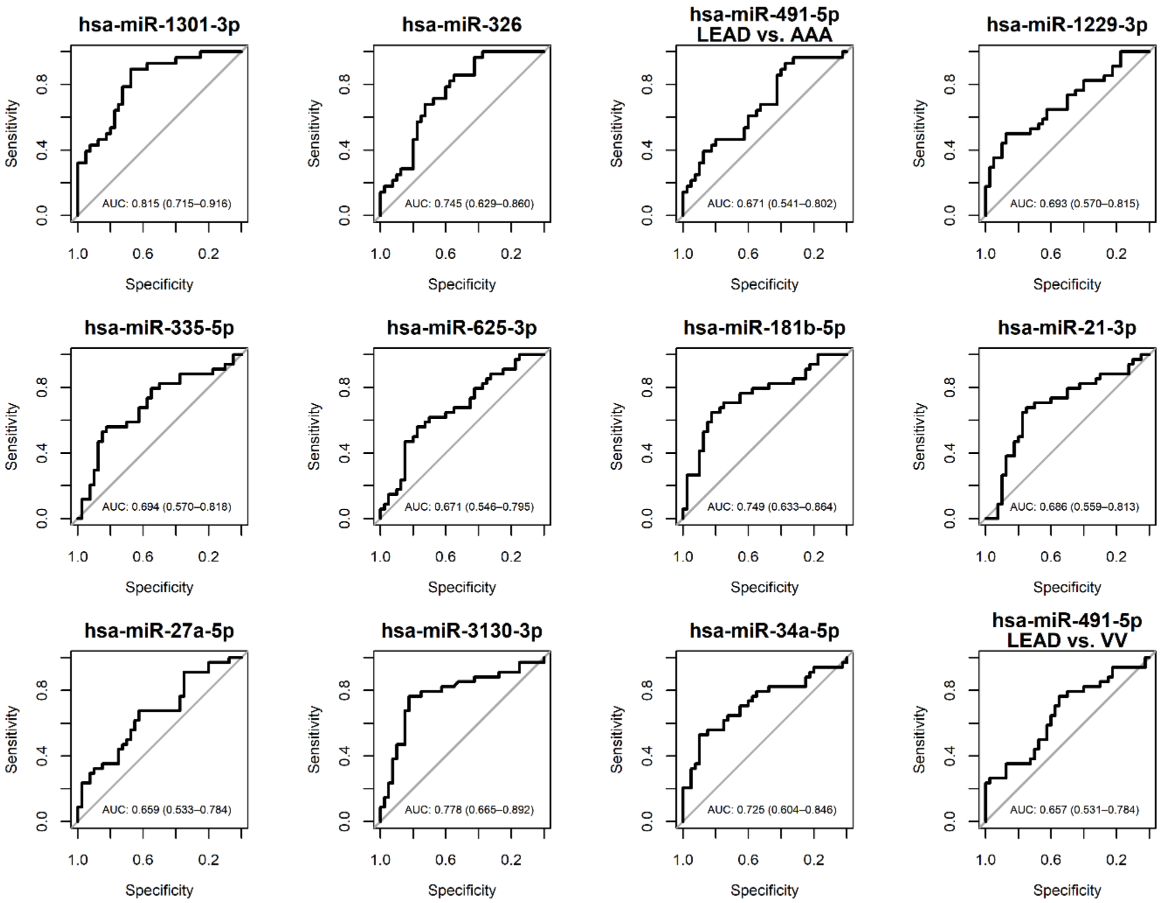

Figure S34. Results of Receiver Operating Characteristics (ROC) analysis performed for selected miRNAs.
